# Human PrimPol is a highly error-prone polymerase regulated by single-stranded DNA binding proteins

**DOI:** 10.1093/nar/gku1321

**Published:** 2014-12-29

**Authors:** Thomas A. Guilliam, Stanislaw K. Jozwiakowski, Aaron Ehlinger, Ryan P. Barnes, Sean G. Rudd, Laura J. Bailey, J. Mark Skehel, Kristin A. Eckert, Walter J. Chazin, Aidan J. Doherty

**Affiliations:** 1 Genome Damage and Stability Centre, School of Life Sciences, University of Sussex, Brighton BN1 9RQ, UK; 2 Departments of Biochemistry and Chemistry and Center for Structural Biology, Vanderbilt University School of Medicine, Nashville, TN 37232, USA; 3 The Jake Gittlen Laboratories for Cancer Research Penn State College of Medicine, 500 University Drive, Hershey, PA 17033, USA; 4 MRC Laboratory of Molecular Biology, Cambridge, CB2 0QH, UK

## Abstract

PrimPol is a recently identified polymerase involved in eukaryotic DNA damage tolerance, employed in both re-priming and translesion synthesis mechanisms to bypass nuclear and mitochondrial DNA lesions. In this report, we investigate how the enzymatic activities of human PrimPol are regulated. We show that, unlike other TLS polymerases, PrimPol is not stimulated by PCNA and does not interact with it *in vivo*. We identify that PrimPol interacts with both of the major single-strand binding proteins, RPA and mtSSB *in vivo.* Using NMR spectroscopy, we characterize the domains responsible for the PrimPol-RPA interaction, revealing that PrimPol binds directly to the N-terminal domain of RPA70. In contrast to the established role of SSBs in stimulating replicative polymerases, we find that SSBs significantly limit the primase and polymerase activities of PrimPol. To identify the requirement for this regulation, we employed two forward mutation assays to characterize PrimPol's replication fidelity. We find that PrimPol is a mutagenic polymerase, with a unique error specificity that is highly biased towards insertion-deletion errors. Given the error-prone disposition of PrimPol, we propose a mechanism whereby SSBs greatly restrict the contribution of this enzyme to DNA replication at stalled forks, thus reducing the mutagenic potential of PrimPol during genome replication.

## INTRODUCTION

Accurate and efficient DNA replication is essential for the maintenance of genomic integrity. The replication machinery is a highly specialized multi-protein complex employed for this purpose, with the replicative DNA polymerases (Pols), Pol α, Pol δ and Pol ϵ, tasked with the majority of bulk DNA synthesis in the eukaryotic nucleus. In mitochondria, this task is undertaken by Polγ. These enzymes are superbly adapted to maximize faithful DNA synthesis, however, this high degree of specialization comes at a cost. Helix-distorting DNA lesions and structures, which persist into the S-phase of the cell cycle, present an obstacle to the replicative polymerases, causing stalling of the replication fork (1). In response, cells employ a variety of DNA damage tolerance mechanisms to facilitate lesion/structure bypass and permit continued replication (2,3).

Mechanisms of replication restart include homologous recombination, in which an alternative sister template permits extension of the stalled primer terminus (2,3). Firing of dormant origins, discontinuous generation of Okazaki fragments on the lagging strand or re-priming of the replication fork downstream of the lesion on the leading strand, can also restart stalled forks (4). An alternative mechanism is translesion DNA synthesis (TLS). Here, specialized DNA polymerases, predominantly of the Y-family, rescue stalled replication forks by directly synthesizing across the damaged nucleotides. In contrast to replicative DNA polymerases, TLS Pols display low fidelity during replication of undamaged DNA templates, thus requiring strict regulation (2). The primary level of regulation for TLS polymerases comes with their inherent distributive character. Additional regulation of access to the replisome is proposed to occur, in part, through post-translational modification of the proliferating cell nuclear antigen (PCNA) (5). Collision of the replication fork with DNA lesions, and consequent stalling, stimulates mono-ubiquitination of PCNA, increasing its affinity for TLS polymerases, thus promoting recruitment of these enzymes to the fork. Following bypass of the lesion, the TLS polymerase dissociates and the high fidelity replicative polymerases proceed with DNA synthesis (5). The polymerase switch, therefore, acts to limit DNA replication by the low fidelity TLS polymerases, permitting access only when lesion bypass is required. Recently, a novel polymerase called PrimPol has been reported to be involved in DNA damage tolerance and TLS during both nuclear and mitochondrial replication (6).

PrimPol is a eukaryotic DNA primase-polymerase, belonging to the archaeo-eukaryotic primase (AEP) superfamily, that undertakes lesion bypass roles in both nuclear and mitochondrial DNA replication (6–10). This enzyme is capable of synthesizing primers using either, nucleoside triphosphates (NTPs) or deoxynucleoside triphosphates (dNTPs), conferring the ability to re-prime and restart replication downstream of DNA lesions. PrimPol also possesses robust template-dependent DNA polymerase activity, which it can utilize to bypass highly distorting pyrimidine 6–4 pyrimidone photoproducts (6–4 PPs) and oxidative lesions, including the common 8-oxo-7,8-dihydrodeoxyguanosine (8-oxo-dG) lesion, thus establishing PrimPol as a competent TLS polymerase (7–10). PrimPol possesses two distinct domains; an enzymatic AEP polymerase domain required for the catalytic activities of the enzyme and a UL52-like zinc finger (Zfn) domain necessary for primase activity and modulating the processivity and fidelity of the enzyme (11). PrimPol knockout (PrimPol^−/−^) cells display increased sensitivity to DNA damaging agents and decreased replication fork rates (7), in addition to defects in mitochondrial DNA (mtDNA) replication (10). Furthermore, PrimPol^−/−^ mouse embryonic fibroblasts have increased metaphase aberrations and chromatid breaks, increasing substantially following low-dose aphidicolin treatment (7). In trypanosomes, deletion of a PrimPol orthologue leads to growth arrest in G_2_ followed by cell death (8). Recent studies have established the involvement of PrimPol in DNA damage tolerance through at least two mechanisms, re-priming and TLS. However, the regulation of PrimPol's contribution to DNA replication has not previously been explored.

In this report, we describe how the enzymatic activities of PrimPol are regulated. We observe that, in contrast to other TLS polymerases, PrimPol does not interact with PCNA and is not stimulated by its presence *in vitro.* Pull-down studies identify that human PrimPol interacts with replication protein A (RPA), the nuclear single-stranded DNA binding protein (SSB), and its mitochondrial equivalent, mitochondrial-SSB (mtSSB). We find that PrimPol interacts with the N-terminal domain of the RPA70 subunit (RPA70N), RPA has previously been shown to stimulate the activity of Pol α and Pol δ (12,13), mtSSB also stimulates its respective polymerase partner, Pol γ (14). However, in stark contrast, we demonstrate that both of these proteins act to significantly limit both the primase and polymerase activities of PrimPol. We demonstrate that PrimPol is an error-prone DNA polymerase, with a strong preference to generate base insertions and deletions, thus necessitating strict regulation during its involvement in DNA synthesis. We propose that SSBs potentially act to limit the contribution of PrimPol to DNA replication in order to limit error-prone synthesis during the bypass of lesions and other genetic obstacles.

## MATERIALS AND METHODS

### Affinity purification of PrimPol complexes for mass spectrometry (MS) analysis

For the large-scale affinity purification of soluble human PrimPol for MS analysis, thirty 175 cm^2^ flasks of confluent Flp-In T-REx-293 cells engineered for PrimPol expression were used, 1 day before harvesting, PrimPol expression was induced in 15 of these flasks by addition of 10 ng/ml doxycycline. Following harvesting and collection, cell pellets (∼1 g each) were lysed in 15 ml lysis buffer (150 mM NaCl, 30 mM Tris pH 7.4, 0.5% NP-40, with Roche protease inhibitor cocktail) and incubated at 4°C on a rocker for 20 min. Input was retained (500 μl) and 1 ml of *Strep*-tactin resin (packed volume) added to the lysate and placed on a rocker for 2 h at 4°C. Washes were also performed in batch mode (with lysis buffer containing 0.1% NP40) and then the *Strep*-tactin resin was transferred to a gravity flow column and washed further. Five successive 500 μl elutions with lysis buffer containing 2 mM desthiobiotin were performed, and each snap frozen with 10% glycerol. Following western blot analysis to determine which affinity purifications were successful, the chosen elutions were concentrated using a VivaSpin 10 000 kDa molecular filter before resolving on a Bis-Tris 4–20% gel and colloidal Coomassie staining (Invitrogen). Whole lane gel extraction was performed with each lane being divided into 1–2 mm bands, which were placed in a 96-well plate before trypsin digestion and MS analysis.

### Mass spectrometry

Polyacrylamide gel slices (1–2 mm) containing the purified proteins were prepared for mass spectrometric analysis using the Janus liquid handling system (PerkinElmer, UK). Briefly, the excised protein gel pieces were placed in a well of a 96-well microtitre plate and destained with 50% v/v acetonitrile and 50 mM ammonium bicarbonate, reduced with 10 mM DTT and alkylated with 55 mM iodoacetamide. After alkylation, proteins were digested with 6 ng/μl Trypsin (Promega, UK) overnight at 37°C. The resulting peptides were extracted in 2% v/v formic acid, 2% v/v acetonitrile. The digest was analysed by nano-scale capillary LC-MS/MS using a Ultimate U3000 high pressure liquid chromatography (ThermoScientific, San Jose, USA) to deliver a flow of ∼300 nl/min. A C18 Acclaim PepMap100 5 μm, 100 μm × 20 mm nanoViper (Thermo Scientific, San Jose, USA), trapped the peptides prior to separation on a C18 Acclaim PepMap100 3 μm, 75 μm × 150 mm nanoViper (Thermo Scientific Dionex, San Jose, USA). Peptides were eluted with a gradient of acetonitrile. The analytical column outlet was directly interfaced via a modified nano-flow electrospray ionization source, with a hybrid linear quadrupolefourier transform mass spectrometer (LTQ Orbitrap XL/ETD, Thermo Scientific, San Jose, USA). Data-dependent analysis was carried out, using a resolution of 30 000 for the full MS spectrum, followed by eight MS/MS spectra in the linear ion trap. MS spectra were collected with an automatic target gain control of 5 × 10^5^ and a maximum injection fill time of 100 ms over a m/z range of 300–2000. MS/MS scans were collected using an automatic gain control value of 4 × 10^4^ and a threshold energy of 35 m/z for collision-induced dissociation. LC-MS/MS data were then searched against a protein database (UniProt KB) using the Mascot search engine programme (Matrix Science, UK) (15). Database search parameters were set with a precursor tolerance of 5 ppm and a fragment ion mass tolerance of 0.8 Da. One missed enzyme cleavage was allowed and variable modifications for oxidized methionine, carbamidomethyl cysteine, pyroglutamic acid, phosphorylated serine, threonine and tyrosine were included. MS/MS data were validated using the Scaffold programme (Proteome Software Inc., USA) (16). All data were additionally interrogated manually.

### Construction of human PrimPol mutants

Human PrimPol and PrimPol^1–487^ were cloned as previously described (11). The 24–354 mutant of PrimPol (PrimPol^24–354^) was constructed by polymerase chain reaction using the following forward and reverse primers; FWD: GTTTCTTCATATGCGGTTGTCCTCAGTGATAGACC, REV: 5′-GTTTCTTGCGGCCGCGATACTGTTAAAATATCCAACC-3′.

### Expression and purification of recombinant proteins

Wild-type PrimPol and PrimPol^24–354^ were expressed in *Escherichia coli* SHuffle Express cells overnight at 16°C, proteins were then purified as previously described (11). Human recombinant PCNA, Pol δ, RPA and mtSSB proteins were expressed and purified as reported previously (17,18). In addition, kTaq-PolA and Tgo-PolB^exo-^ were purified as previously described (19,20). Protein concentrations were determined based on absorbance at 280 nm corrected with the protein-specific extinction coefficient. Extinction coefficient values for each of the recombinant proteins were calculated using ProtParam tool (ExPASy). Phage T4 SSB and T4 polymerase were purchased from New England Biolabs. Polγ^exo−^ was a kind gift from Dr Whitney Yin (University of Texas, USA).

### Electrophoretic mobility shift assays (EMSAs)

EMSAs were performed for 60 min at room temperature in 20 μl volumes containing 50 mM Potassium acetate, 20 mM Tris-acetate pH 7.9, 10 mM Magnesium acetate, 1 mM DTT, 60 nM single-stranded fluorescein labelled DNA (sequence 16, Supplementary Table S1) and varying concentrations of mtSSB or RPA (as indicated on Supplementary Figure S1). Reactions were supplemented with 2 μl 25% (w/v) Ficoll and resolved on a 5% (v/v) native polyacrylamide gel at 75 V for 60 min in 0.5× TBE buffer. Gels were scanned using a FujiFilm FLA-5100 image reader.

### Nuclear magnetic resonance (NMR) methods

RPA70N and RPA32C were expressed and purified as described previously (21,22). ^15^N-^1^H HSQC experiments were performed at 25°C on a Bruker Avance III 800 MHz NMR spectrometer with a cryogenically cooled probe. Spectra were acquired for samples of ^15^N-enriched RPA32C or RPA70N alone and in the presence of full-length PrimPol or PrimPol^1–487^. All samples were equilibrated in a buffer containing 20 mM HEPES, 80 mM NaCl, 2 mM DTT and 5% deuterium oxide.

### DNA primase assays

DNA primase assays were performed using the previously described protocol (11), in buffer containing 50 mM Potassium acetate, 20 mM Tris-acetate pH 7.9, 10 mM Magnesium acetate and 1 mM DTT. The templating oligonucleotide sequence can be found as sequence 10 in Supplementary Table S1. In assays containing SSBs, reactions were supplemented with 4 μM mtSSB, 8 μM T4 SSB or 8 μM RPA, before the addition of PrimPol. Note that twice as much RPA than mtSSB was used due to the increased level of RPA required to completely shift the DNA probe in EMSA reactions (Supplementary Figure S1). An excess of SSBs over single-stranded DNA was used to ensure that the template was fully coated, taking into account the binding site size of the protein and the length of the ssDNA binding interface. Reaction products were resolved on a 15% (v/v) polyacrylamide gel containing 7 M urea and 1× TBE buffer at 850 V for 2.5 h in 1× TBE buffer. Gels were scanned with a FujiFilm FLA-5100 image reader.

### DNA primer extension assays

Primer extension assays were performed using 5′ Hexa-chlorofluorescein-labelled DNA primers (ATDbio) (sequences 1–4 in Supplementary Table S1) annealed to complementary unlabelled DNA templates (sequences 5–15 in Supplementary Table S1). Extensions were carried out at 37°C in 20 μl volumes containing; 100 nM of the assayed polymerase (or 3U/ml of T4 Pol), 20 nM primer-template substrate, 100 μM dNTPs (NEB), 50 mM Potassium acetate, 20 mM Tris-acetate pH 7.9, 10 mM Magnesium acetate, 1 mM DTT and 2 μg bovine serum albumin (BSA; NEB). In the case of single nucleotide incorporation assays, 100 μM of the individual dNTP to be assayed was added in place of all dNTPs. For assays using SSBs, DNA templates were pre-incubated on ice with 200 nM mtSSB, 400 nM RPA, or 400 nM T4 SSB, before the addition of enzymes. Again, twice as much RPA than mtSSB was used due to the apparent lower affinity of RPA for DNA as perceived in EMSA experiments (Supplementary Figure S1). Extension reactions were monitored over varying time courses, typically 1, 3, 5 and 10 min (except where indicated), and quenched with 20 μl stop buffer (95% formamide, 0.05% bromophenol blue, 0.09% xylene cyanol and 200 nM competitor oligonucleotide). Products were boiled at 95°C for 5 min before resolution on a 15% (v/v) polyacrylamide/ 7 M urea gel. Gels were scanned using an FLA-5100 image reader (Fujifilm). Primer extension products were quantified using ImageQuant TL software (GE Life Sciences).

### The pSJ4 plasmid-based *lacZα* fidelity assay

The fidelity of human PrimPol was determined using the pSJ4 plasmid-based *lacZα* reporter gene assay. The pSJ4 plasmid is a customised version of the previously described pSJ3 plasmid (23). The practical feature of the pSJ4 plasmid is a short 64 nt long gap (versus 163 nt long gap of pSJ3), which is more efficiently filled up by distributive DNA polymerases *in vitro*. This specific feature was necessary to implement in the pSJ4 plasmid to make it suitable for measurements of fidelity of human PrimPol. Typical pSJ4 gap filling reaction was carried out in a total volume of 10 μl comprising: reaction buffer (50 mM Potassium acetate, 20 mM Tris-acetate pH 7.9, 10 mM Magnesium acetate, 1 mM DTT and 2 μg BSA), 20 fmol of gapped pSJ4 plasmid, 100 μM of each dNTP and 50 nM PrimPol. The gap filling reaction was carried out at 37°C for 30 min and completion was confirmed using an analytical digestion with EcoRI (Fermentas) restriction endonuclease followed by 1% agarose electrophoresis. As a control the pSJ4 *lacZα* reporter gene assay was used to measure the fidelity of two well-characterized thermostable DNA polymerases, the Klenow fragment kTaq-PolA and Tgo-PolB^exo−^. The fidelity of both of the thermostable Pols was measured as described previously (23).

### *In vitro* HSV-*tk* mutagenesis Assay

Primed single-stranded DNA and gapped-duplex substrates were prepared as previously described (24,25). Primer-extension reactions were initiated with 1.6 μM PrimPol in buffer containing 10 mM Bis Tris Propane-HCl pH 7.0, 10 mM MgCl_2_, 1 mM DTT, 500 μM dNTPs and 200 nM primed ssDNA substrate in 50 μl total volume. Reactions were terminated after 15 min. Enzyme was used in excess conditions due to PrimPol's distributive synthesis pattern and ssDNA binding capacity. The 81 nucleotide HSV = tk target sequence was isolated by MluI and StuI digestion and hybridised to a gapped heteroduplex DNA molecule. After confirming hybridization by agarose gel electrophoresis, FT334 *E. coli* (*upp, tdk*) were transformed with hybridised DNA. Transformed bacteria were plated on VBA plates containing 50 μg/ml chloramphenicol (Cm) in the absence or presence of 40 μM 5-fluoro-2′-deoxyuridine (FUdR) to determine HSV-tk mutation frequencies, as described (25). The observed HSV-TK frequency is calculated as the ratio of FUdR^R^/Cm^R^ to Cm^R^ colonies, and was determined for three independent reactions. DNA sequence analysis was conducted on independent mutants isolated from two independent PrimPol reactions per template. The polymerase error frequency (Pol EF) was calculated by subtracting the ssDNA background mutation frequency from the HSV-TK frequency. To correct the Pol EF for HSV-tk mutants with multiple polymerase errors, the *Pol EF*_est_ was derived as described (26), using the following formula:

\begin{equation*} Pol\;EF_{est} = Pol\;EF/\sum\limits_{n = 1}^3 {\frac{1}{n}\left( {\frac{{mutants\;with\;n\;errors}}{{total\;mutants\;analyzed}}} \right)} \end{equation*}

where *n* is the total number of detectable errors that were >10 nucleotides apart within the same sequence.

## RESULTS

### PrimPol is not stimulated by PCNA

**Figure 1.**
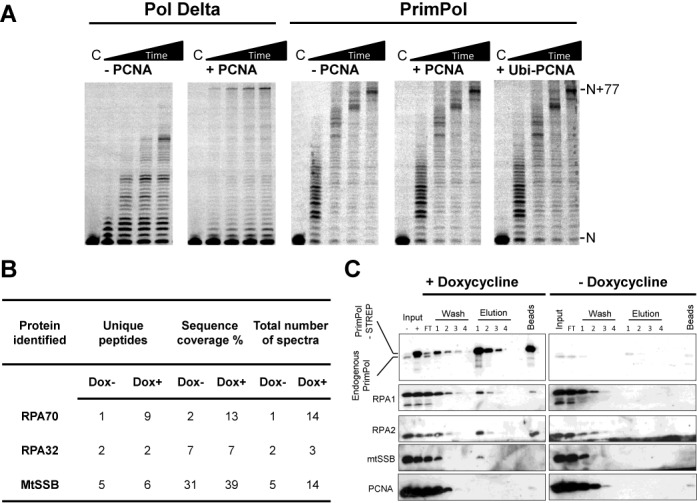
PrimPol is not regulated by PCNA but does interact with SSBs. (A) PrimPol or Polδ (100 nM) were incubated with primer template substrates (20 nM) and dNTPs (100 μM) at 37ºC for increasing times (1, 3, 5, 10 min) in the absence or presence of PCNA (200 nM). PrimPol's primer extension activity was not stimulated in the presence of either unmodified or mono-ubiquitinated PCNA. In contrast Polδ shows increased processivity when PCNA is present. ‘C’ indicates the no enzyme control. (B) Identification of binding partners of PrimPol as analysed through MS analysis, showing enrichment of RPA subunits 1 and 2, and mtSSB. The fold enrichment of each protein is indicated on the right of the table. (C) Western blot validation of PrimPol interacting proteins identified in the MS analysis. PrimPol co-purifies with both mtSSB and RPA, but not with PCNA. Flp-In T-Rex-293 cells engineered for inducible expression of PrimPol^FlagStrep^ were grown in the presence or absence of doxycycline (10 ng/ml, 24 h) and PrimPol^FlagStrep^ was affinity purified from the soluble lysate using *Strep*-Tactin resin. Fractions from the affinity purification were analysed by western blot with the indicated antibodies. Input ‘+’ and ‘-’ represent the clarified lysate of cells grown in the presence or absence of doxycycline, respectively.

Mono-ubiquitination of PCNA in response to DNA damage increases its affinity for TLS polymerases, such as Pol η, Pol κ, Pol ι and REV1, promoting their recruitment to the replication fork in order to facilitate lesion bypass (27). *In vitro* studies have shown that the ability of Pol η and REV1 to bypass an abasic site is stimulated by the presence of mono-ubiquitinated PCNA over unmodified PCNA. However, on an undamaged template, PCNA stimulates the polymerase activities of these enzymes to a similar degree, regardless of its ubiquitination status (28). In order to investigate whether PrimPol is stimulated by PCNA, we assessed the impact of both unmodified and mono-ubiqutinated PCNA on the polymerase activity of the enzyme *in vitro*. To do this, we employed primer extension assays on a 97-mer DNA template (sequence 9, Supplementary Table S1) annealed to a 20 nucleotide primer (sequence 2, Supplementary Table S1). Unlike the stimulating effect of PCNA on the polymerase activity of Pol η and REV1, we find that both unmodified and mono-ubiquitinated PCNA have no stimulatory effect on the polymerase activity of PrimPol (Figure 1A). In contrast, the presence of unmodified PCNA increased the processivity of Pol δ in the same conditions (Figure 1A). These results demonstrate that PrimPol, unlike other TLS polymerases, is not stimulated by either unmodified or mono-ubiquitinated PCNA, suggesting that the enzyme is regulated by another distinct mechanism.

### PrimPol interacts with RPA and mtSSB *in vivo*

To identify cellular factors that associate with PrimPol, and thus may be involved in regulating this polymerase *in vivo*, we purified PrimPol from cultured human cells and identified co-purifying proteins using MS. To facilitate affinity purification of PrimPol, we fused it to a *Strep*-tag, which exploits the high affinity and specific binding between streptavidin and its natural ligand biotin (29). Specifically, the eight amino acid long *Strep*-Tag II (WSHPQFEK) was used, which allows affinity purification with the streptavidin derivative *Strep*-Tactin and specific elution with desthiobiotin (29,30). The Flp-In T-REx system was used to generate a stable cell line with doxycycline-inducible expression of *Strep*-tagged PrimPol. Affinity purified *Strep*-tagged PrimPol and co-purifying proteins were resolved by sodium dodecyl sulphate-polyacrylamide gel electrophoresis and excised gel bands analysed by MS.

Proteins identified in the MS analysis were ranked according to percentage of total spectra in the induced sample, and the fold enrichment calculated for each. A large set of proteins (1249) were identified, of these ∼550 were present only in the induced sample and a further 65 showed a >3-fold enrichment, with PrimPol enriched 20-fold. Input of these proteins into the Database of Annotation, Visualization, and Integrated Discovery clustered these proteins into a number of functionally related groups (31,32). Consistent with the dual localization of PrimPol, two of the predominant groups were nuclear and mitochondrial proteins. A large proportion of DNA and nucleotide binding proteins were also present, and more specifically proteins involved in DNA replication and repair, such as RPA (Figure 1B). In contrast, no mitochondrial replication enzymes were present, although mtSSB was enriched.

To validate the potential PrimPol interacting proteins from the preliminary MS analysis, small-scale affinity purifications of *Strep*-tagged PrimPol from whole cell lysate were performed and analysed by western blot. Following addition of doxycycline a predominant species of ∼69 kDa was detected by western blot analysis with an anti-PrimPol antibody (Figure 1C), and furthermore, endogenous PrimPol was also detected, with little *Strep*-tagged PrimPol visible in the non-induced lysate (Figure 1C). Analysis of the affinity purification with RPA70 and RPA32 antibodies, and the mitochondrial equivalent, mtSSB, after stringent washing, all gave specific bands in the elutions (Figure 1C), indicating that PrimPol associates with these proteins. The RPA findings agree with recent studies by Wan *et al.* (9), which reported that PrimPol interacts with RPA and that this interaction may be required for recruitment of PrimPol to stalled replication forks (9). ATAD3, a mitochondrial membrane-associated ATPase and core nucleoid component, was also detected in the elutions, however, ATAD3 did appear to bind to the *Strep*-tactin resin in the non-induced sample (data not shown). Notably, analysis of the affinity purification using an anti-PCNA antibody did not give detectable bands in the elutions (Figure 1C), suggesting that, unlike other TLS polymerases, PrimPol does not interact with PCNA. This result agrees with the inability of PCNA to stimulate PrimPol and further suggests that PrimPol is not regulated by PCNA *in vivo*. Although many potential interactions were identified by MS, we have validated that both major classes of cellular single-stranded DNA binding protein (RPA and mtSSB) co-purify with PrimPol, while PCNA does not.

### RPA70N protein recruitment domain of RPA mediates interaction with PrimPol

In order to cross-validate the interaction between RPA and PrimPol, as well as characterise the domains responsible, we screened the two primary RPA protein recruitment domains DBD-N of RPA1 (RPA70N) and the winged-helix domain of RPA2 (RPA32C) using NMR spectroscopy. To this end, ^15^N-^1^H Heteronuclear Single Quantum Coherence (HSQC) NMR spectra of ^15^N-enriched RPA70N^1–120^ and RPA32C^172–270^ were acquired in the absence and presence of 2-fold molar excess of unlabelled PrimPol. These spectra monitor amide chemical shifts, which are sensitive to their local chemical environment. Thus, binding of a ligand is expected to perturb the location and/or intensity of the peaks from residues at the binding site. We note that additional chemical shift perturbations can occur within globular protein interaction domains as a result of structural changes induced by ligand binding.

**Figure 2.**
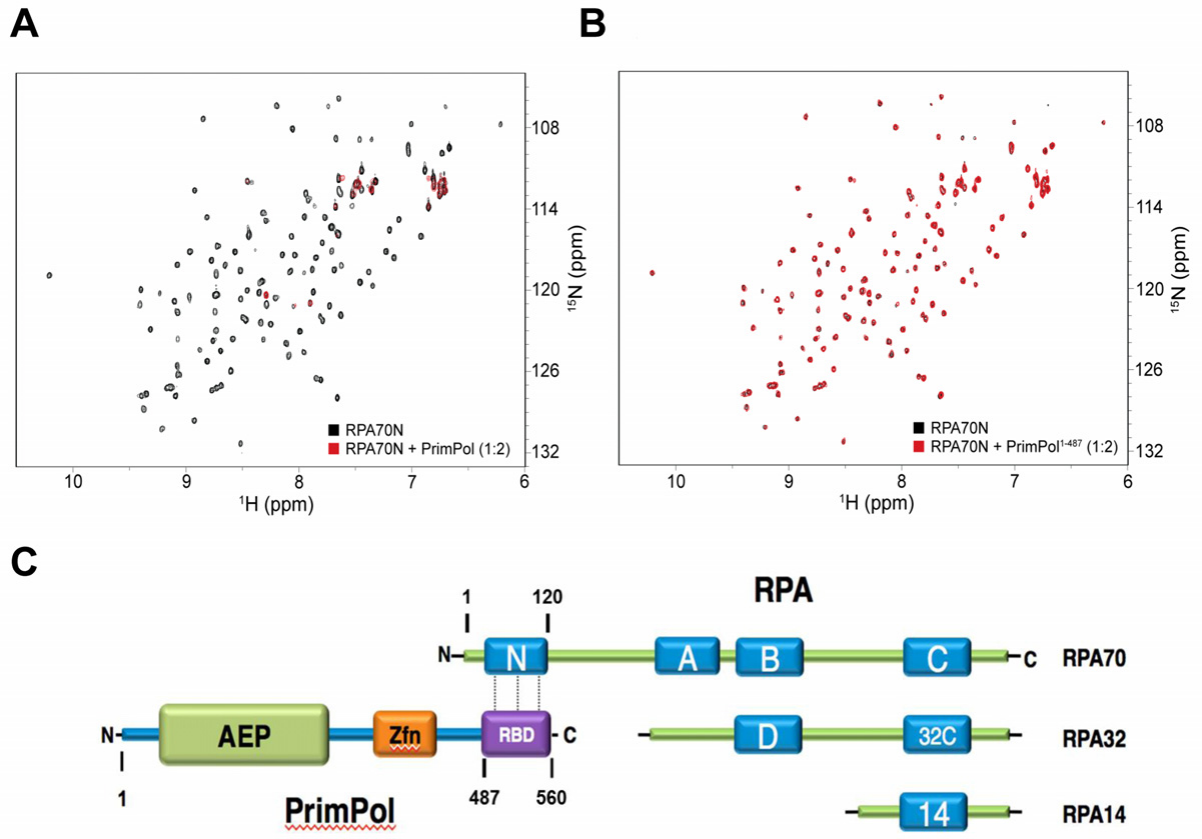
Characterization of the RPA-PrimPol domain interactions. ^15^N-^1^H HSQC overlays of 50 μM ^15^N-enriched RPA70N alone (A and B, black) and in the presence of 100 μM of PrimPol (A, red) or PrimPol^1–487^ (B, red). All spectra were obtained as 25°C in a buffer containing, 20 mM HEPES, 80 mM NaCl, 2 mM DTT and 5% deuterium oxide at pH 7.1. Schematic showing the RBD of PrimPol and the RPA70N domain of RPA where it binds (C).

The NMR analysis of the interaction of RPA32C with PrimPol revealed no significant chemical shift perturbations (Supplementary Figure S2). In contrast, addition of PrimPol to RPA70N generated substantial perturbations (Figure 2A). The primary effect observed is loss of signal intensity for the majority of peaks (Figure 2A, red), which we attribute to the large increase in mass as the ∼13 kDa protein tumbles much more slowly when part of the ∼78 kDa complex. These observations indicate that RPA70N serves as the primary PrimPol interaction site on RPA.

Having mapped the primary interaction domain of RPA, we next used NMR to better define the interaction region of PrimPol. Previous immunoprecipitation data suggested that the C-terminal region of PrimPol is responsible for its interaction with RPA (9). We therefore examined the binding to RPA70N of a PrimPol deletion construct lacking the C-terminal 73 residues (PrimPol^1–487^). Figure 2B shows that the HSQC spectrum of RPA70N with PrimPol^1–487^ closely resembled that of the free protein. Thus, loss of the C-terminal region causes PrimPol to lose its ability to bind to RPA70N. Together, these results support a model in which the primary interaction between PrimPol and RPA is mediated by the RPA70N and PrimPol^488–560^ domains (Figure 2C).

### RPA and mtSSB suppress *de novo* primer synthesis by PrimPol

**Figure 3.**
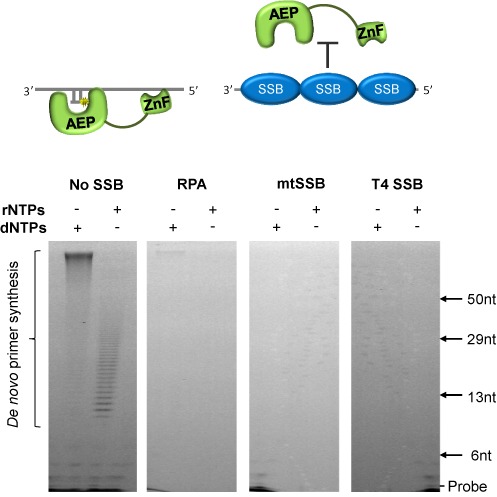
The effect of SSBs on the primase activity of human PrimPol. Single-stranded poly-dT templates (500 nM) were incubated with dNTPs or rNTPs (500 μM) and human PrimPol (1 μM), either alone or in the presence of RPA (8 μM), mtSSB (4 μM) or T4 SSB (8 μM) for 1 h at 37ºC (see ‘Materials and Methods’ for details). In the absence of SSBs PrimPol is capable of *de novo* primer synthesis using either dNTPs or rNTPs. However, when templates are coated with SSBs PrimPol is unable to synthesize primers. The schematic above represents how this inhibition is likely a result of RPA and mtSSB blocking PrimPol binding sites on the ssDNA.

Recent studies identifying that PrimPol's C-terminal RPA-interacting domain is required for foci formation and appropriate functioning of the enzyme *in vivo*, suggest that RPA may act to recruit PrimPol to the replication fork (9). However, the effect of RPA on the enzymatic activities of PrimPol has not previously been studied. To determine the effect of both RPA and mtSSB on the primase activity of PrimPol, we analysed the enzyme's ability to synthesize primers on a 60-mer poly-dT ssDNA template (sequence 10, Supplementary Table S1), coated with either RPA or mtSSB. T4 SSB coated ssDNA was also included as a non-PrimPol-interacting control. As observed previously (7,11), PrimPol facilitated primer synthesis on the ssDNA template in the absence of SSBs. However, RPA and mtSSB strongly inhibited the ability of PrimPol to synthesize primers, both with dNTPs and rNTPs (Figure 3). PrimPol also failed to synthesize primers on ssDNA coated with T4 SSB. This suggests that SSBs supress the primer synthesis ability of PrimPol by blocking potential DNA binding sites for the enzyme. These findings echo previous studies of the Polα complex, which demonstrated that primer synthesis was suppressed on ssDNA templates coated with RPA (33).

### RPA and mtSSB impede primer extension by PrimPol

In contrast to the suppression RPA imposes on Pol α during primer synthesis, RPA stimulates the polymerase and processivity activities of the enzyme when elongating primers (12). This implies that RPA acts to prevent Pol α binding to ssDNA, hence negating primer synthesis but actively promotes primer extension. Furthermore, RPA and mtSSB have been shown to stimulate the polymerase activities of Pol δ and Pol γ, respectively (13,14). Therefore, we next investigated the effect of RPA and mtSSB on the polymerase extension activity of PrimPol. T4 SSB was again included as a non-interacting control. In order to investigate this, we employed a standard primer extension assay in the presence of RPA, mtSSB or T4 SSB. This represents a physiologically relevant situation in which the replication fork has stalled at a site of DNA damage leading to uncoupling of the stalled replicative polymerase and the MCM helicase, resulting in the generation of long stretches of SSB-bound ssDNA (34). In addition to full-length PrimPol, we also analysed a truncation of the enzyme (PrimPol^24–354^) that lacks both the ssDNA binding ZnF domain and the C-terminal region required for RPA interaction. This truncation allowed investigation into the effect of SSBs on the AEP domain of PrimPol alone, which has previously been shown to possess polymerase activity (11).

**Figure 4.**
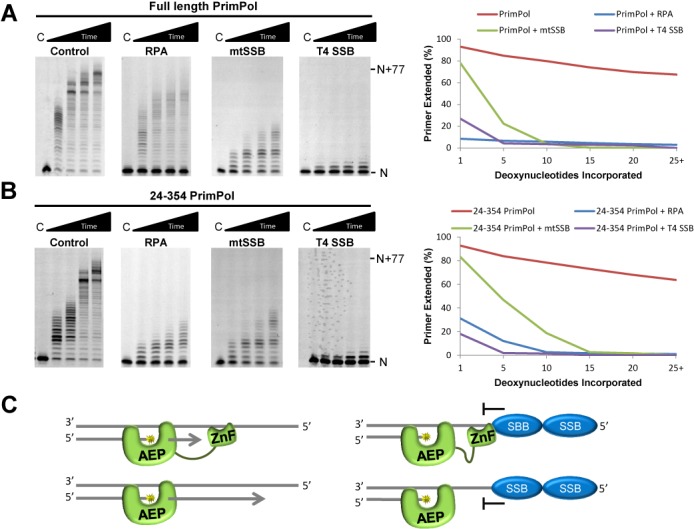
The effect of SSBs on the primer extension activity of PrimPol. (A) Primer-template substrates (20 nM) and dNTPs (100 μM) were incubated with PrimPol (100 nM), either alone or with RPA (400 nM), mtSSB (200 nM) or T4 SSB (400 nM), for increasing times (1, 3, 5, 10 min). In the presence of SSBs, full-length PrimPol's primer extension activity is severely impeded. The primer runs at the position indicated ‘*N*’, with full extension denoted by ‘*N* + 77’. ‘C’ indicates the no enzyme control. For each gel, the 10-min time-point was quantified to identify the percentage of primers extended more than 1, 5, 10, 15, 20 or 25 bases. This quantification is shown on the right of the gels. (B) The primer extension activity of PrimPol^24–354^ is also restricted in the presence of SSBs. Quantification of the 10-min time-point for each gel is again shown to the right of the gels. (C) Schematic representation of the inhibitory effect of SSBs on the primer extension activity of PrimPol.

In the absence of SSBs, the full-length PrimPol and PrimPol^24–354^ fully extended the majority of primers by the final time point (Figure 4A and B). However, the presence of RPA, mtSSB or T4 SSB, dramatically impeded primer extension by both enzymes (Figure 4A and B). This inhibition caused both a reduction in the length of extended primers and an increase in the amount of unextended DNA substrate. The partial extension observed in the presence of SSBs suggests that PrimPol was unable to displace these proteins from the template DNA during primer extension. As a result of the dynamic nature of SSBs binding to DNA, any ssDNA unbound by SSBs that is close to the primer-template junction would be available for extension by PrimPol until the enzyme was restricted by SSBs bound downstream or dissociated and was unable to bind again due to exclusion by SSB. The varying levels of inhibition observed in the presence of different SSBs may therefore be a result of the different binding footprints and affinities of the SSBs used, in conjunction with the length of the template. The inhibition of PrimPol^24–354^, coupled with the inhibitory effect of T4 SSB on full-length PrimPol, indicates that this effect is not due to an interaction between PrimPol and the SSBs. Furthermore, inhibition of PrimPol^24–354^ suggests that the inhibitory effect of SSBs is not only due to competition with PrimPol's ZnF domain for binding of ssDNA (Figure 4B).

In order to ensure that the inhibitory effect of RPA and mtSSB on PrimPol primer-extension is not simply a result of the amount of protein used, we repeated the assay in the presence of a large range of SSB concentrations. In each case, a similar level of inhibition was observed at protein concentrations sufficient to coat the single-stranded region of the DNA template (Supplementary Figures S3 and S4). A similar level of inhibition was also observed when PrimPol was pre-incubated with the template before adding dNTPs and SSBs (Supplementary Figure S5). In addition, Pol δ with PCNA, Pol γ^exo−^ and T4 Pol were able to displace RPA, mtSSB and T4 SSB, respectively, and fully extend the primer in the same conditions in which PrimPol activity is limited (Supplementary Figure S6). This reveals a striking difference in the ability of PrimPol to displace SSBs in comparison to replicative polymerases.

Stimulation of Pol γ by mtSSB has previously been shown to be salt-dependent, maximally stimulated at 20 mM potassium chloride and inhibited at concentrations ∼100 mM (14). We tested whether the inhibition of PrimPol by RPA and mtSSB was also salt-dependent by repeating the primer extension assays in a range of salt concentrations. The restraining impact of RPA and mtSSB on the polymerase activity of PrimPol was consistent across the range of salt concentrations tested, between 0 and 60 mM KCl (Supplementary Figures S7 and S8), ruling out the possibility that this effect is salt-dependent.

RPA has previously been implicated in modulating the fidelity of 8-oxo-dG bypass by Pol λ and Pol η, specifically acting to increase accurate dCTP incorporation opposite the lesion (35). We have recently reported that PrimPol is able to bypass 8-oxo-dG lesions by incorporation of either dCTP or dATP. In addition, PrimPol incorrectly incorporates dTTP opposite the first T of a 6–4 photoproduct and incorporates dATP opposite deoxyuracil, while the full-length enzyme is unable to bypass CPDs or abasic sites in magnesium (11). We examined whether RPA or mtSSB affected the ability, or fidelity, of lesion bypass across a range of different templating lesions (sequences 11–15, Supplementary Table S1) using single incorporation primer extension assays with the lesion immediately downstream of the primer-template junction. RPA and mtSSB did not appear to alter either the ability to bypass lesions or the fidelity of this bypass, except in the case of the 6–4 photoproduct where bypass was inhibited in both cases (Supplementary Figure S9). This may be due to the shorter length of the 6–4 photoproduct template compared to the length of the other lesion-containing templates (sequences 11–15 in Supplementary Table S1). Alternatively, the SSBs might prevent the looping-out mechanism which has been proposed to be employed by PrimPol for bypass of 6–4 photoproducts (36).

These results demonstrate that PrimPol's polymerase activity is severely limited in the presence of SSBs. PrimPol has been confirmed as a competent TLS polymerase with roles in DNA damage tolerance *in vivo* (6). As such, PrimPol is likely to be recruited to stalled replication forks, possibly by RPA (9), where it will encounter long stretches of RPA/mtSSB-bound ssDNA, a result of uncoupling of the replicative polymerase and helicase. Therefore, the inability of PrimPol to displace SSBs during primer elongation likely acts as a mechanism to limit PrimPol's contribution to DNA replication. In order to identify the necessity to restrict DNA synthesis by PrimPol during genome replication, we next investigated the fidelity and mutagenicity of this enzyme.

### PrimPol shows a propensity for misincorporation and mispair extension

**Figure 5.**
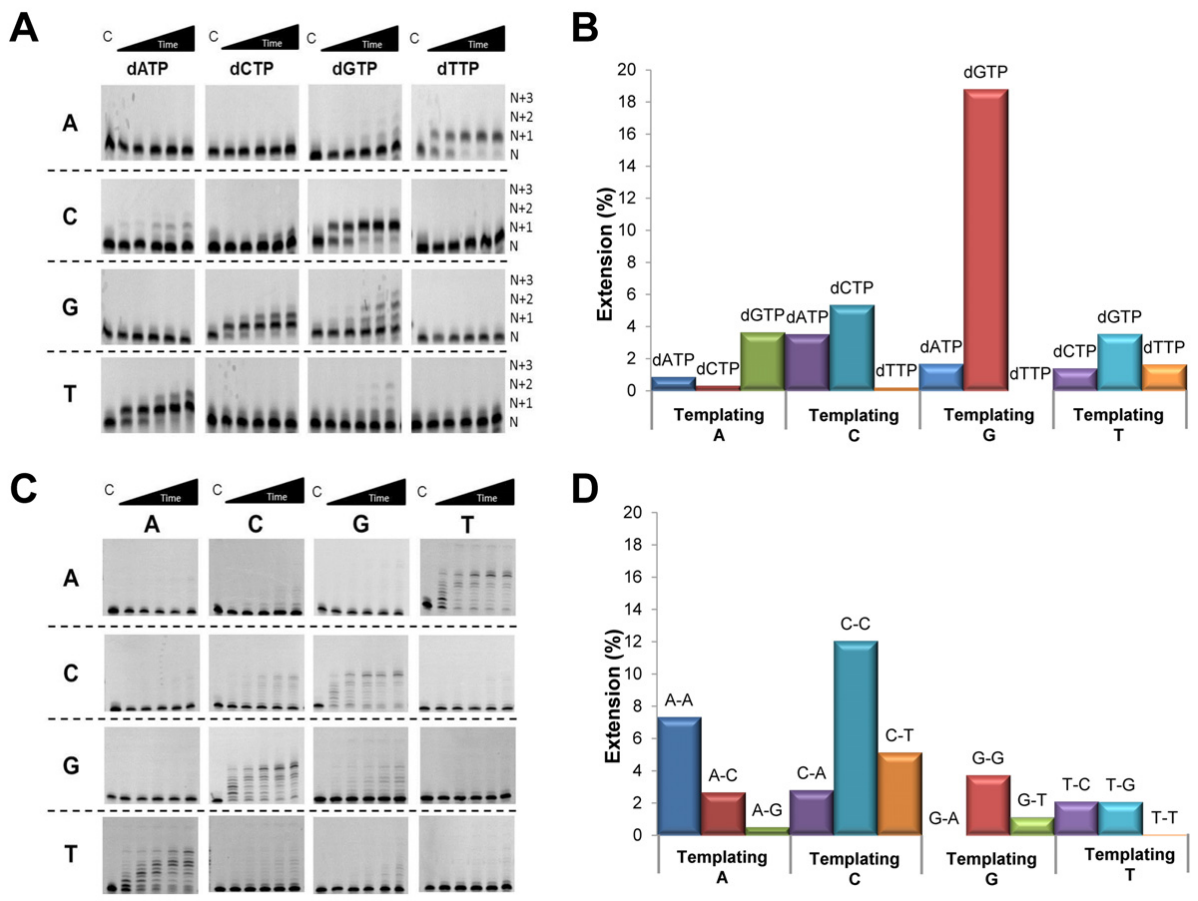
PrimPol can misincorporate bases and extend from mismatched bases. (A) Analysis of misincorporation by PrimPol. PrimPol (100 nM) was incubated at 37ºC with primer-template substrates (20 nM) containing either A, C, G or T, as the templating base immediately downstream of the primer for increasing times (1, 3, 5, 10 min). Individual reactions contained either dATP, dCTP, dGTP or dTTP (100 μM). The templating bases are indicated on the left, while the dNTP provided is shown above. Dotted lines separate reactions where the templating base is the same. ‘*N*’ denotes the position at which the primer runs whilst ‘*N* + 1’, ‘*N* + 2’ and ‘*N* + 3’ indicate incorporation of 1, 2 or 3, bases, respectively. (B) Quantification of the misincorporation assays at each 10-min time-point. Data were normalized against the correct incoming base. (C) Analysis of mismatch extension by PrimPol. PrimPol was incubated at 37ºC for increasing times (1, 3, 5, 10 min) in the presence of all four dNTPs and primer-template substrates containing a mismatched base at the 3′ end of the primer The templating base for each gel is indicated on the left, while the corresponding mismatched primer base is shown above. (D) Quantification of the mismatch extension assays at each 10-min time-point. Data were normalized against extension from correctly matched bases.

As an initial investigation into the base substitution fidelity of PrimPol, we used a primer extension assay based on single incorporation of either the correct or incorrect incoming base (Figure 5A). PrimPol was incubated with a short primer-template with either adenine (A), cytosine (C), guanine (G) or thymine (T), as the immediate templating base (*N* + 1 position). This base was followed by two templating Cs (*N* + 2 and *N* + 3 positions), except where C was the base at *N* + 1, in which case A and G were the upstream templating bases (*N* + 2 and *N* + 3 positions). For each primer-template substrate, the reaction was supplemented with only one of the four dNTPs (dATP, dCTP, dGTP or dTTP). Quantification of these data and normalization to correct incorporation suggest that PrimPol has a strong propensity to misincorporate dGTP, especially opposite a templating G (Figure 5B). However, when dGTP is the incoming base, significant product bands were visible at the *N* + 2 and *N* + 3 positions (Figure 5A). This increased *N* + 2 and *N* + 3 incorporation could result from PrimPol misaligning the *N* + 1 templating base, rather than through misincorporation, which would in turn suggest a potential to generate base deletions.

PrimPol also showed a preference to misincorporate dCTP and dATP opposite a templating C (Figure 5A and B). Consistently, a significant *N* + 2 product was visible on the G template when dCTP was the incoming base (Figure 5A). Again, this corresponds to misincorporation of dCTP opposite a templating C at the *N* + 2 position. A similar result was observed on the T template with the correct incoming base (dATP), indicating misincorporation of dATP opposite a templating dC at the *N* + 2 position (Figure 5A). These results suggest that PrimPol has a propensity to misincorporate both dCTP and dATP opposite a templating C, which may be a potential error signature of PrimPol.

We also analysed the ability of PrimPol to extend different terminal mismatched base pairs (Figure 5C and D). PrimPol was particularly proficient at extending C-C mismatches, with ∼12% of the primers being extended relative to extension of a correctly matched C-G primer-template junction. The enzyme also showed a capacity to extend A-A, C-T, G-G and A-C/C-A mismatches, while showing a much lower ability to extend from T-T and G-A/A-G mismatches (Figure 5C and D). Together, these data reveal that PrimPol is able to facilitate both misincorporation of bases and perform extension of these base mispairs.

### PrimPol is an error-prone polymerase with a preference to generate base insertions and deletions

Unlike the major human replicative polymerases (Pol δ and Pol ϵ), PrimPol lacks a 3′ to 5′ exonuclease proofreading domain and is therefore expected to be a potentially error-prone DNA polymerase. To characterise PrimPol's error frequency and spectrum, we employed a plasmid based *lacZ*α reporter gap-filling assay (23). Due to the distributive nature of PrimPol we modified the recently developed pSJ3 plasmid to create a new plasmid (pSJ4) containing a shorter 64-nt-long gapped region. Initially, the fidelity of two well characterised DNA polymerases (Klenow fragment of Taq-Pol A and exonuclease-deficient variant of Tgo-Pol B^exo−^ was measured. From raw mutation frequencies, an absolute error rate (number of mistakes made per base incorporated) was calculated as previously described (23). The Klenow fragment Taq-Pol A and Tgo-Pol B^exo−^ presented error rates of 3.6 × 10^−5^ and 1.6 × 10^−5^, respectively (Table 1), agreeing with the previously reported fidelities of these enzymes (23,37). Analysis of human PrimPol revealed an error-prone phenotype with a calculated error rate of 1 × 10^−4^, essentially an order of magnitude lower than the exonuclease-deficient variants of *S. cerevesiae* replicative DNA polymerases δ, ϵ and the TLS specialized DNA polymerase ζ (38–40).

**Table 1.** Mutation frequencies observed for the Klenow fragment Taq-Pol, Tgo-Pol^exo−^ and human Prim-Pol enzymes

Polymerase	Total number of colonies^a^	Number of white (mutant) colonies	Corrected mutation frequency^b^	Error rate^c^
Klenow Taq-Pol A	58 555	96	1.6 × 10^−3^	3.6 × 10^−5^
Tgo-Pol B (exo^−^: D215A)	48 167	33	0.7 × 10^−3^	1.6 × 10^−5^
Prim-Pol	54 667	264	4.6 × 10^−3^	1.0 × 10^−4^

The fidelity measurements were determined using plasmid-based gap filling assay (pSJ4-*lacZα*). The pSJ4 plasmid assay was developed to study fidelity of translesion DNA polymerases and it is modified version of previously described pSJ3 plasmid (23).

^a^The fidelity of each polymerase was determined in three separate experiments, each of which involved scoring *E. coli* colonies on nine separate plates. The aggregated numbers are given.

^b^Corrected mutation frequency equals:

({number of white colonies/total number of colonies} – background mutation rate). A background mutation rates of 1.7 × 10^−5^ were used for gapped pSJ4.

^c^Error rate is the number of mistakes made per base incorporated. The corrected mutation frequency was converted to error rate as previously described (23).

An expression frequency (*P*) of 0.3 was used. Due to limited amount of sequencing data set Ni/N value of 1 was used and the number of detectable sites (D) was the sum of the number of determined base substitutions plus insertion/deletions, that is, 147 for pSJ4.

**Figure 6.**
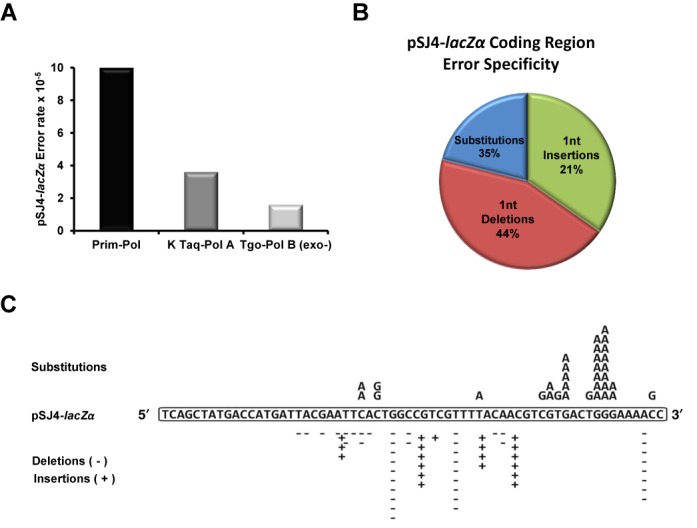
Mutational signature of human PrimPol and the *lacZα* sequence. (**A**) The average pSJ4*-lacZα* coding region error rate of PrimPol, relative to other polymerases. The fidelity of each polymerase was determined in three separate experiments, each of which involved scoring *E. coli* colonies on 9 separate plates. The aggregated numbers are given. (**B**) Proportions of base substitutions, insertions and deletions generated in the pSJ4*-lacZα* coding region. Pie chart depicts percentages, from 95 total mutational events observed. (**C**) The diagram shows the 64-nt-long sequence of the *lacZα* reporter synthesized by human PrimPol *in vitro* in the pSJ4 gap-filling fidelity assay. A total of 95 white colonies were sequenced revealing the unique mutation signature of human PrimPol.

The mutations generated by PrimPol while copying the 64-nt-long fragment of the *lacZα* reporter are visualized in Figure 6. PrimPol exhibited a 10-fold preference for base substitution mutations when C or G were the templating bases, in comparison to errors introduced when copying A or T. Perhaps more intriguing, however, was the finding that more than half of the mutations observed were base deletions/insertions, rather than the expected base substitutions (Figure 6B; Table 2). This apparent propensity of PrimPol to generate a very high proportion of insertion-deletion (indel) errors seems to support the previously proposed template scrunching mechanism, which PrimPol can employ to skip damaged nucleobases (36).

**Table 2.** Individual changes introduced into the *lacZα* indicator gene of pSJ4 by human Prim-Pol are shown

Mutation Type	Number	Frequency (%)
A→T/T→A	1	1.05
A→C/T→G	2	2.1
A→G/T→C	0	0
G→A/C→T	24	25.3
G→C/C→G	4	4.2
G→T/C→A	2	2.1
Insertions	20	
Deletions	42	44.2
Total	95	100
A→N/T→N	3	3.16
G→N/C→N	30	31.6
Transitions	24	25.3
Transversions	9	9.5

A total of 95 white colonies were completely sequenced in order to reveal the actual types and frequency of mutations.

To investigate this phenomenon further and cross-validate the findings we also measured the fidelity of PrimPol using the HSV-tk forward mutation assay (25). We engineered the HSV-tk gene substrates to contain additional short tandem repeat (STR) sequences within the 5′ region of the gene, in order to study polymerase fidelity within repetitive sequences. Using [T]_8_ and [A]_8_ STR-containing substrates, the observed HSV-tk frequency resulting from PrimPol DNA synthesis is 1400 ± 360 × 10^−4^ and 900 ± 210 × 10^−4^, respectively (Table 3). In comparison to the replicative polymerases α and δ, PrimPol displays a 16 to- 28-fold increase in HSV-tk frequency (Supplementary Table S2) (24,41). Furthermore, PrimPol displays an error frequency that is more than 2-fold higher than the repair and specialized polymerases η, κ and β.

**Table 3.** PrimPol error rates within STR and HSV-tk coding sequences

Mutational Target	Frequency x 10^−4^	
	T_8_	A_8_
Observed HSV-tk frequency ± SD^a^	1400 ± 360	900 ± 210
ssDNA Background	0.7	0.7
Pol EF_est_^b^	1300 (59)^c^	770 (40)
STR Region	470 (21)	210 (11)
HSV-tk Coding Region	860 (38)	560 (29)
Frameshifts	590 (26)	380 (20)
Large Deletions	110 (5)	100 (5)
Complex	160 (7)	80 (4)
Base Substitutions	<23 (0)	<19 (0)

^a^Mutant frequencies are mean ± standard deviation of three independent reactions.

^b^Pol EF_est_ is calculated as described in methods.

^c^Number of independent errors from two reactions.

**Figure 7.**
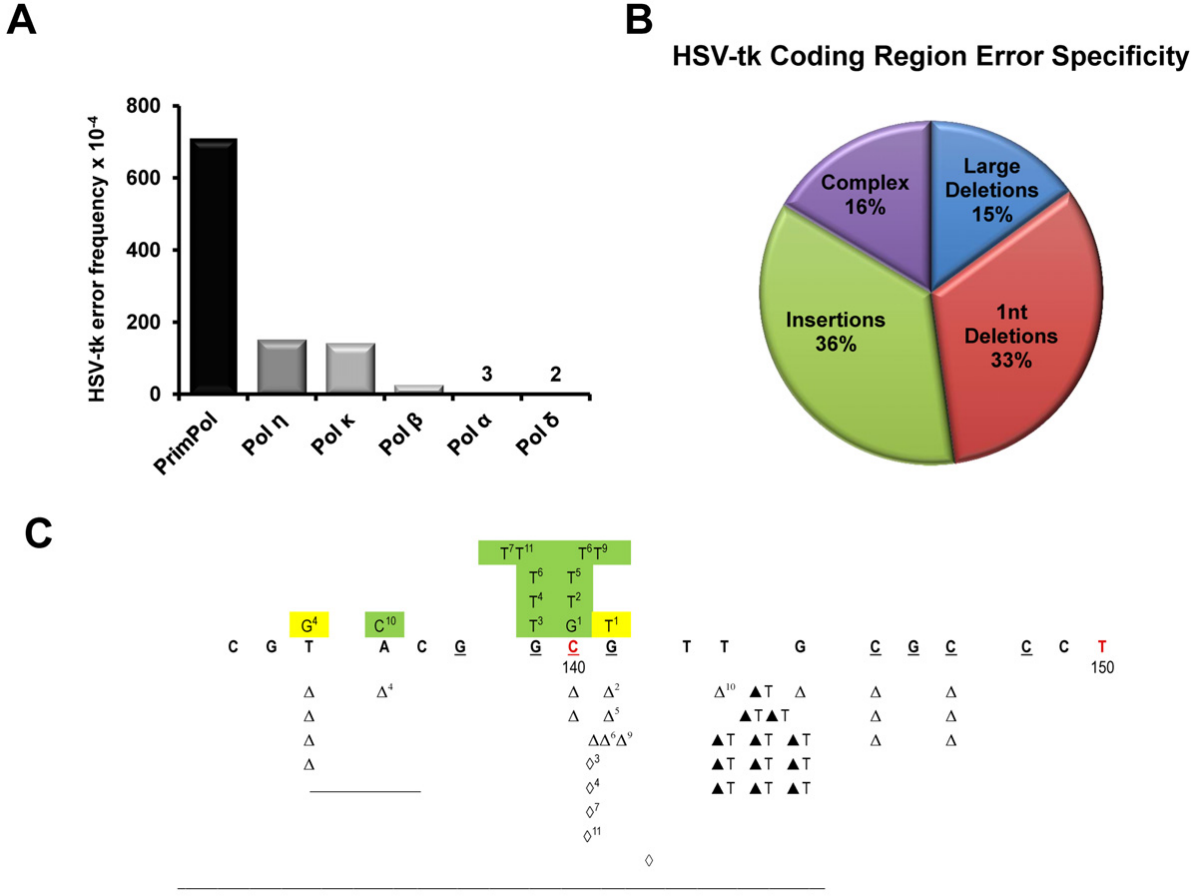
Human PrimPol's replication of the HSV-tk coding region is error-prone and unique. (**A**) The average HSV-tk coding region error frequency of PrimPol, relative to other human polymerases. Data generated from (24,41), and Supplementary Table S2. (B) Types of errors created by PrimPol made in the HSV-tk coding region. Pie chart depicts percentages, from 67 total mutational events observed. (**C**) A mutation hot-spot in the coding region is shown to highlight PrimPol's unique error specificity. Base substitutions are shown above the template sequence, and are highlighted green for phenotypically detectable events or yellow for non-detectable events. Single deletion and insertion events are shown below the template with open and closed triangles, respectively, while diamonds indicate a tandem deletion. Superscripts mark the errors found within an individual complex event. The underlined bases within the template denote the sequence with hairpin-forming potential.

PrimPol creates errors within the artificial STR sequence at a rate comparable to Pols η, κ and β (Supplementary Table S2). Additionally, PrimPol's error specificity within the STR region is in-line with what we have previously observed for other polymerases at these repeats (Supplementary Figure S10), suggesting that STR errors are not what is driving PrimPol's low fidelity. In contrast to the STR region, PrimPol's error frequency within the coding-region of the HSV-tk gene is higher than any other polymerase analysed to date (Figure 7A). In agreement with the *lacZ*α gap-filling assay, PrimPol's coding region error spectrum is almost entirely insertion/deletion (indel) based, with a bias towards deletions (Figure 7B; Supplementary Figure S11). The very high proportion of insertion errors (36%) is a phenotype never observed in this assay for other DNA polymerases, and is tremendously different from error-prone Pol η which creates indel frameshift and base-substitution errors at similar rates (41). Although we have observed Pol κ insertion errors, the vast majority of Pol κ indels were deletions (42) and all of the Pol β indel errors we have observed in the HSV-tk gene were deletion events (25). These findings show that PrimPol has an error specificity unique amongst DNA polymerases.

We observed a pronounced mutational hotspot that included both indels and complex errors within a sequence that can potentially from a hairpin structure (Figure 7C). Both the complex events and the insertion events result in changes to the template sequence that expand the [T]_2_ template sequence to a [T]_3_ or [T]_4_ sequence. Intriguingly, while such an observation would suggest PrimPol is prone to expand repeated sequences, only a single expansion event was observed at both the [T]_8_ and [A]_8_ STR sequences (Supplementary Figure S10).

## DISCUSSION

PrimPol is a recently discovered primase-polymerase that is important for translesion synthesis during DNA replication in eukaryotic cells. Unlike other TLS enzymes, PrimPol does not interact with, nor is it stimulated by, unmodified or mono-ubuitinated PCNA, indicating that other factors potentially regulate its activities *in vivo*. Nevertheless, our results do not rule out the possibility that PrimPol might interact with PCNA indirectly through an additional bridging partner. In this report, we identify a potential regulatory mechanism employed to limit the contribution of PrimPol to DNA replication that is distinct from that used by other TLS polymerases. This mechanism involves SSBs that directly restrict DNA synthesis by PrimPol by limiting the availability of ssDNA template, downstream of the stalled replisome, thus preventing re-binding of PrimPol.

It was recently reported that PrimPol interacts with RPA1 and that this interaction is required for foci formation *in vivo* (9). These initial studies suggested that RPA may be involved in the recruitment/regulation of PrimPol at sites of DNA damage. Here, the RPA binding domain (RBD) of PrimPol, in conjunction with the ssDNA binding ZnF domain, may act as a docking mechanism for recruitment of the enzyme. This would allow tethering of PrimPol to stretches of ssDNA partially coated with RPA, following stalling of the replication fork. In this report, we further explored the interaction between PrimPol and SSBs, in addition to the impact that these proteins have on the activity of PrimPol *in vitro.* We identified that PrimPol interacts with mtSSB, as well as RPA (Figure 1C), pertaining to the role of the enzyme in mitochondrial, as well as nuclear, DNA replication (7,10). Furthermore, we establish that PrimPol interacts with the RPA70N protein recruitment domain (Figure 2). This is in contrast to previous reports suggesting that PrimPol interacts with RPA70C (9). This revision is consistent with the absence of any other published data suggesting RPA70C is involved in interactions with other proteins. However, it remains possible that PrimPol may have two different sites of interaction on the RPA70 subunit.

It is surprising that both RPA and mtSSB, in addition to the non-interacting T4 SSB, act to significantly impede the primase and polymerase activities of PrimPol (Figures 3 and 4). Previously, RPA has been shown to suppress the ability of the Polα complex to synthesize primers, identifying a role for RPA in preventing non-specific priming events (33). Our results establish that this role also extends to regulating priming by PrimPol, with mtSSB fulfilling an analogous role in mitochondria. Interestingly, this suggests that PrimPol is only able to synthesize primers at regions of the genome not occupied by SSBs, either where SSBs have been displaced by other replication factors, or where the topology of the DNA template prevents SSB binding, for example, where DNA secondary structures occur.

**Figure 8.**
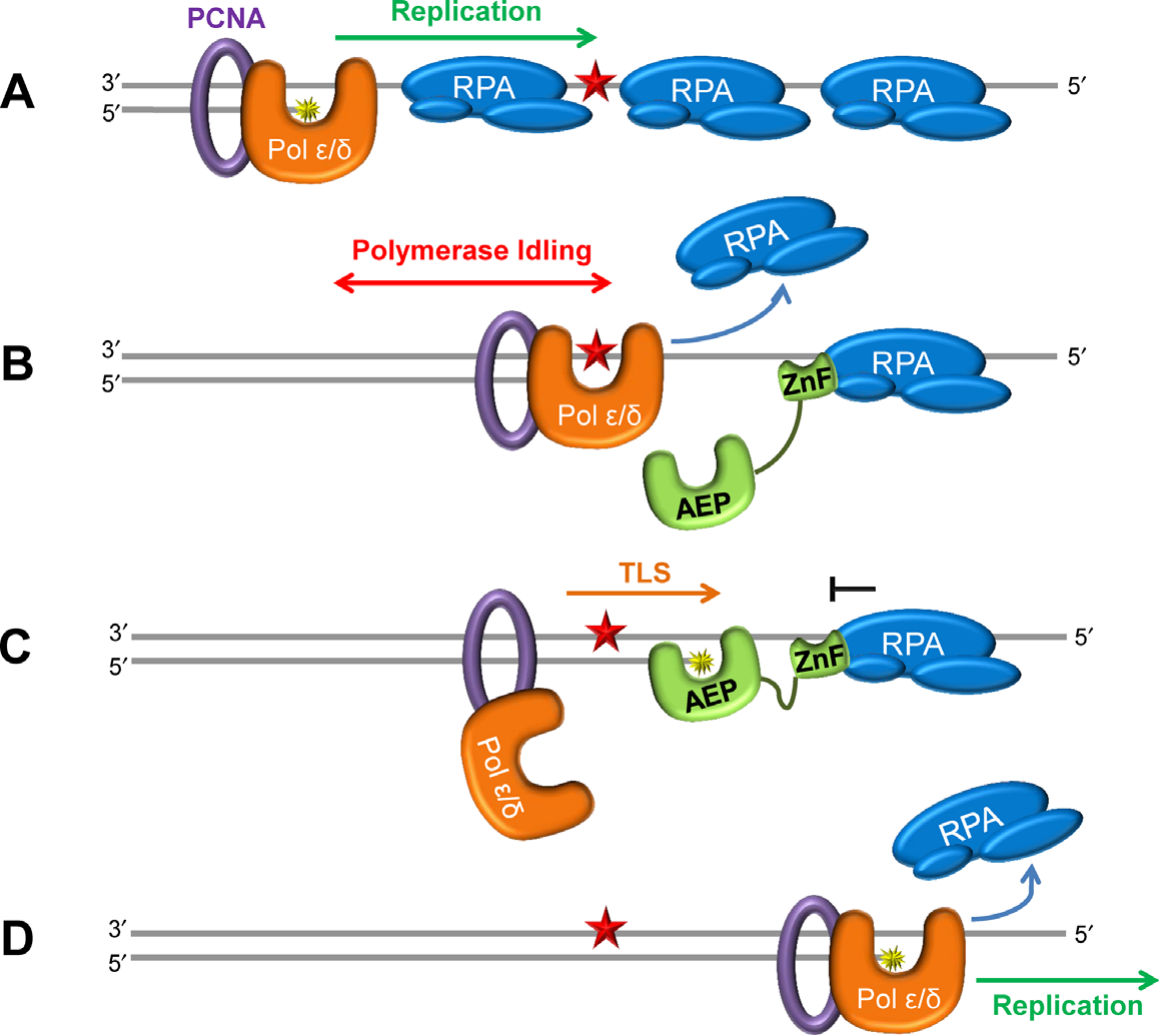
Model for regulation of PrimPol synthesis by SSBs during DNA replication. (**A**) Unperturbed replication proceeds up to the lesion on the SSB-bound ssDNA template, with the replicative DNA polymerase displacing the bound SSB as it synthesizes the daughter DNA strand. (**B**) Replication continues until a lesion is encountered, here the intolerant replicative polymerase stalls at the lesion and idles upstream, displacing any surrounding SSBs and generating a ssDNA interface. This allows recruitment of PrimPol to the downstream SSB, with additional binding to the exposed ssDNA interface through the ZnF domain. (**C**) PrimPol utilizes its AEP domain to catalyse a TLS reaction, here the primer terminus is extended over the lesion before further synthesis is prevented by the downstream SSB. (**D**) Following bypass of the lesion, the replicative polymerase again proceeds with replication.

Previous studies have shown that RPA can stimulate the polymerase activity of Polα and Polδ (13,43), with mtSSB acting to stimulate Polγ (14). In stark contrast, our results show that both RPA and mtSSB severely restrict the polymerase activity of PrimPol (Figure 4). Interestingly, in *E. coli*, SSB inhibits the progression of the TLS polymerase, Pol II (44), and additionally, Pol IV when the interaction between the two proteins is disrupted (45). We have previously shown that PrimPol displays very low processivity, incorporating only ∼4 bases per binding event, suggesting that the enzyme is only required for very short stretches of DNA synthesis (11). Notably, the ZnF domain of PrimPol, which only binds ssDNA, is involved in modulation of the enzymes processivity (11). This domain is believed to be spatially separated from the polymerase domain, fulfilling a role in regulating both the primase and polymerase activities of PrimPol. This distributive nature of PrimPol likely acts as the primary level of regulation to limit the involvement of PrimPol in DNA synthesis. Indeed, the limiting effect of SSBs on PrimPol's polymerase activity may be in part due to the prevention of rebinding of the enzyme to ssDNA, following its dissociation as a result of its low processivity. However, interestingly, we also find that the 24–354 truncation of PrimPol, lacking the ssDNA binding ZnF domain, is also inhibited by SSBs. These results suggest that, in addition to the low processivity of PrimPol, RPA and mtSSB contribute to restraining the enzyme to limit its potentially mutagenic DNA synthesis during replication restart. However, it is possible that additional remodelling factors may permit synthesis by PrimPol on SSB-coated DNA *in vivo*. The contrasting effects of SSBs on replicative polymerases and PrimPol do, however, suggest a potential mechanism of regulation represented by a model summarized in Figure 8.

The stimulatory effect of SSBs on the progression of replicative polymerases permits DNA synthesis on SSB-coated DNA until a lesion is encountered on the template strand (Figure 8A). The intolerant replicative polymerase stalls at the lesion and idles upstream, as a result of its 3′–5′ exonuclease activity (46), consequently displacing any surrounding SSBs and generating a ssDNA interface for access of PrimPol (Figure 8B). PrimPol is then recruited to the SSB bound immediately downstream of the lesion via its SSB-binding domain, additionally binding the exposed ssDNA interface through its ZnF domain. Subsequently, PrimPol utilizes its AEP domain to perform a TLS reaction, extending the primer terminus over the DNA lesion, before further extension is prevented by the downstream SSB (Figure 8C). Bypass of the lesion allows replication to proceed, with the previously stalled replicative polymerase continuing extension (Figure 8D).

Importantly, DNA synthesis by PrimPol is limited by SSBs which likely act to ensure that the enzyme only participates in the synthesis of short stretches of DNA. This level of regulation may be necessary due to the mutagenic potential of PrimPol. We provide experimental evidence that PrimPol is a highly error-prone DNA polymerase. Strikingly, in two forward mutation assays, PrimPol shows a strong propensity to indel errors. Within the HSV-tk sequence, PrimPol created indel errors almost exclusively, with base insertions accounting for more than one-third of PrimPol's error spectrum (Figure 7B). We observed a unique mutational hot-spot for PrimPol (Figure 7C). This region is rich with [CG] repeats, and contains several sequences with the potential to form hairpin structures. The single base insertion events can be explained by classic primer strand misalignment, while the complex events are more difficult to dissect. However, the common result of both insertion and complex errors within this hotspot is the expansion [T]_2_ to [T]_3_, suggesting that the PrimPol complex errors are generated primarily within the loop of the hairpin. PrimPol can displace the template strand when copying sequences with microhomology (36). Possibly, at the HSV-tk hotspot sequence, PrimPol is forced to slip both the primer and template strand to get through the hairpin. While this mechanism is speculative, the data we present do confirm that PrimPol's error specificity is unique from other human polymerases. Together, our observations support the template scrunching mechanism, which PrimPol can employ to skip damaged nucleobases (36). Initially, the scrunching mechanism was observed in the presence of manganese ions during translesion bypass of abasic sites and ultraviolet damage lesions (36). Our data suggest that the same scrunching mechanism is utilized by this enzyme when copying non-damaged DNA, in the presence of magnesium ions.

Therefore, PrimPol's *modus operandi* during synthesis appears to be a double-edged sword, facilitating replication restart at bulky lesions (e.g. 6–4 photo-products) but potentially introducing base insertions and deletions into undamaged templates. This threat to genomic integrity requires tight regulation of the activity of PrimPol during DNA replication and we propose that PrimPol's inability to displace SSBs at the replication fork ensures that the mutagenic potential of this enzyme is greatly limited. In addition, nuclear PrimPol is active primarily in S-phase (36). Intermolecular proofreading of PrimPol synthesis products by either Pols δ or ϵ could limit mutagenesis. Similarly, in mitochondria, pol γ, which has a very active exonuclease domain, could correct PrimPol's errors. Finally, the error-prone bypass of lesions by PrimPol produces a structure that is readily detected by the post-replicative mismatch repair machinery. Clearly, PrimPol's important role in replication and the prevention of chromosomal instability must be balanced with its potential mutagenic activity.

## SUPPLEMENTARY DATA

Supplementary Data are available at NAR Online.

SUPPLEMENTARY DATA
