# Supplementary material for: Human PrimPol is a highly error-prone polymerase regulated by single-stranded DNA binding proteins
Source: Nucleic Acids Res. 2014 Dec 29;43(2):1056–68. doi: 10.1093/nar/gku1321 (PMC4333378; doi:10.1093/nar/gku1321)
Supplement: SUPPLEMENTARY DATA [file supp_43_2_1056__index.html]

Human PrimPol is a highly error-prone polymerase regulated by single-stranded DNA binding proteins — SUPPLEMENTARY DATA 

# Human PrimPol is a highly error-prone polymerase regulated by single-stranded DNA binding proteins

## SUPPLEMENTARY DATA

**Files in this Data Supplement:**

- SUPPLEMENTARY DATA
