# Supplementary material for: Human PrimPol is a highly error-prone polymerase regulated by single-stranded DNA binding proteins
Source: Nucleic Acids Res. 2014 Dec 29;43(2):1056–68. doi: 10.1093/nar/gku1321 (PMC4333378; doi:10.1093/nar/gku1321)
Supplement: SUPPLEMENTARY DATA [file supp_gku1321_nar-03179-h-2014-File010.pdf]

Table S1

#	Oligonucleotide	Label	Sequence
1	HP-16 Primer	5'-Hex	5′-CACTGACTGTATGATG-3′
2	HP-20 Primer	5'-Hex	5′-TGTCGTCTGTTCGGTCGTTC-3′
3	HP-27 Primer	5'-Hex	5′-TGTCGTCTGTTCGGTCGTTCGGTCTTC-3′
4	HP-28 Primer	5'-Hex	5′-TGTCGTCTGTTCGGTCGTTCGGTCTTCA-3′
5	ND-50 $Template_{TT}$	None	5′-CGCGCAGGGCGCACAACAGCCTTGAAGACCGAACGACCGAACAGACGACA-3′
6	ND-50 $Template_{AA}$	None	5′-CGCGCAGGGCGCACAACAGCCAAGAAGACCGAACGACCGAACAGACGACA-3′
7	ND-50 $Template_{CC}$	None	5′-CGCGCAGGGCGCACAACAGAGCCGAAGACCGAACGACCGAACAGACGACA-3′
8	ND-50 $Template_{GG}$	None	5′-CGCGCAGGGCGCACAACAGCCGGGAAGACCGAACGACCGAACAGACGACA-3′
9	ND-97 Template	3'-Biotin	5′- ACCGCGAACTTGAATTCTAGTTCAGTCTAAATGCTCTCAAGCACTGAGCAATTCACAACATATGGCTTT CGATTACCGAACGACCGAACAGACGACA-3′
10	Poly(dT)$_{60}$	5'-Biotin	5′-TTTTTTTTTTTTTTTTTTTTTTTTTTTTTTTTTTTTTTTTTTTTTTTTTTTTTTTTTTTT-3′
11	6-4(PP) Template	None	5′- CTCGTCAGCATCT^TCATCATACAGTCAGTG-3′
12	CPD Template	None	5′-CGCGCAGGGCGCACAACAGCCT=TGAAGACCGAACGACCGAACAGACGACA-3′
13	8-oxo-G Template	None	5′-CGCGCAGGGCGCACAACAGCC8-oxo-GTGAAGACCGAACGACCGAACAGACGACA-3′
14	dUracil Template	None	5′-CGCGCAGGGCGCACAACAGCCUTGAAGACCGAACGACCGAACAGACGACA-3′
15	AP Template	None	5′-CGCGCAGGGCGCACAACAGCCAPTGAAGACCGAACGACCGAACAGACGACA-3′
16	ND-50 ssDNA Template	5'-FAM	5′-CGCGCAGGGCGCACAACAGCCTTGAAGACCGAACGACCGAACAGACGACA-3′

Supplementary Table 1: Sequences of the DNA oligonucleotides used in primase and primer extension assays. Lesions within the sequences are denoted in red.

Table S2

Polymerase	Pol EF_{est} x 10^{-4}		
	Overall	HSV-tk Coding Region	STR Region
PrimPol			
T_8 Template	1300 (59)[a]	860 (38)	470 (21)
A_8 Template	770 (40)	560 (29)	210 (11)
Pol η[b,c]			
T_8 Template	440 (35)	210 (17)	230 (18)
A_8 Template	270 (67)	87 (22)	180 (45)
Pol κ[c]			
T_8 Template	460 (49)	140 (15)	320 (34)
Pol α[b]			
T_8 Template	47 (77)	1.8 (3)	45 (74)
A_8 Template	55 (77)	4.3 (6)	51 (71)
Pol δ[c]			
T_8 Template	33 (32)	2.1 (2)	31 (30)
Pol β[b]			
T_8 Template	380 (83)	32 (7)	350 (76)
A_8 Template	400 (82)	15 (3)	390 (79)

[a] Number of independent errors from two reactions

[b] Published data from Ananda et al., 2014

[c] Published data from Hile et al., 2012

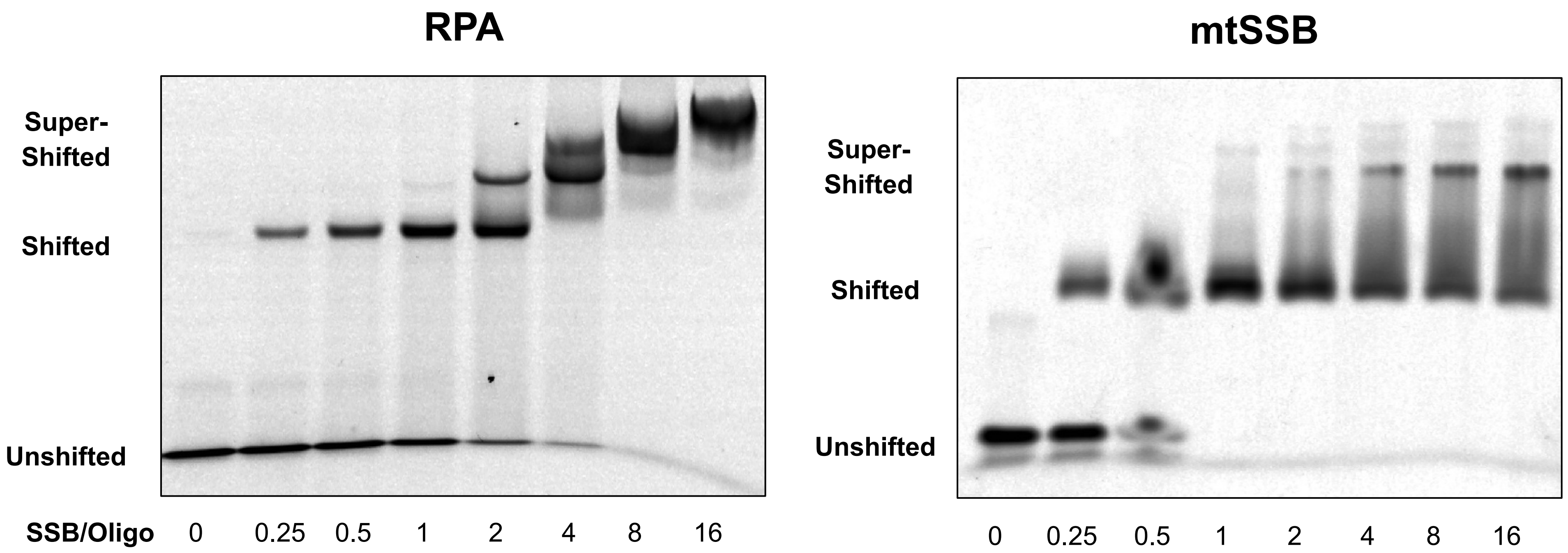

Fig. S1. EMSA experiments for confirmation of RPA and mtSSB ssDNA binding

'SSB/Oligo' below each gel indicates the molar ratio of SSB to the ssDNA template. Note that more than twice as much RPA, compared to mtSSB, was required to fully shift the ssDNA template. Super-shifted bands indicate multiple SSBs bound to the ssDNA template, demonstrating that a large excess of protein over DNA is required to fully coat the template.

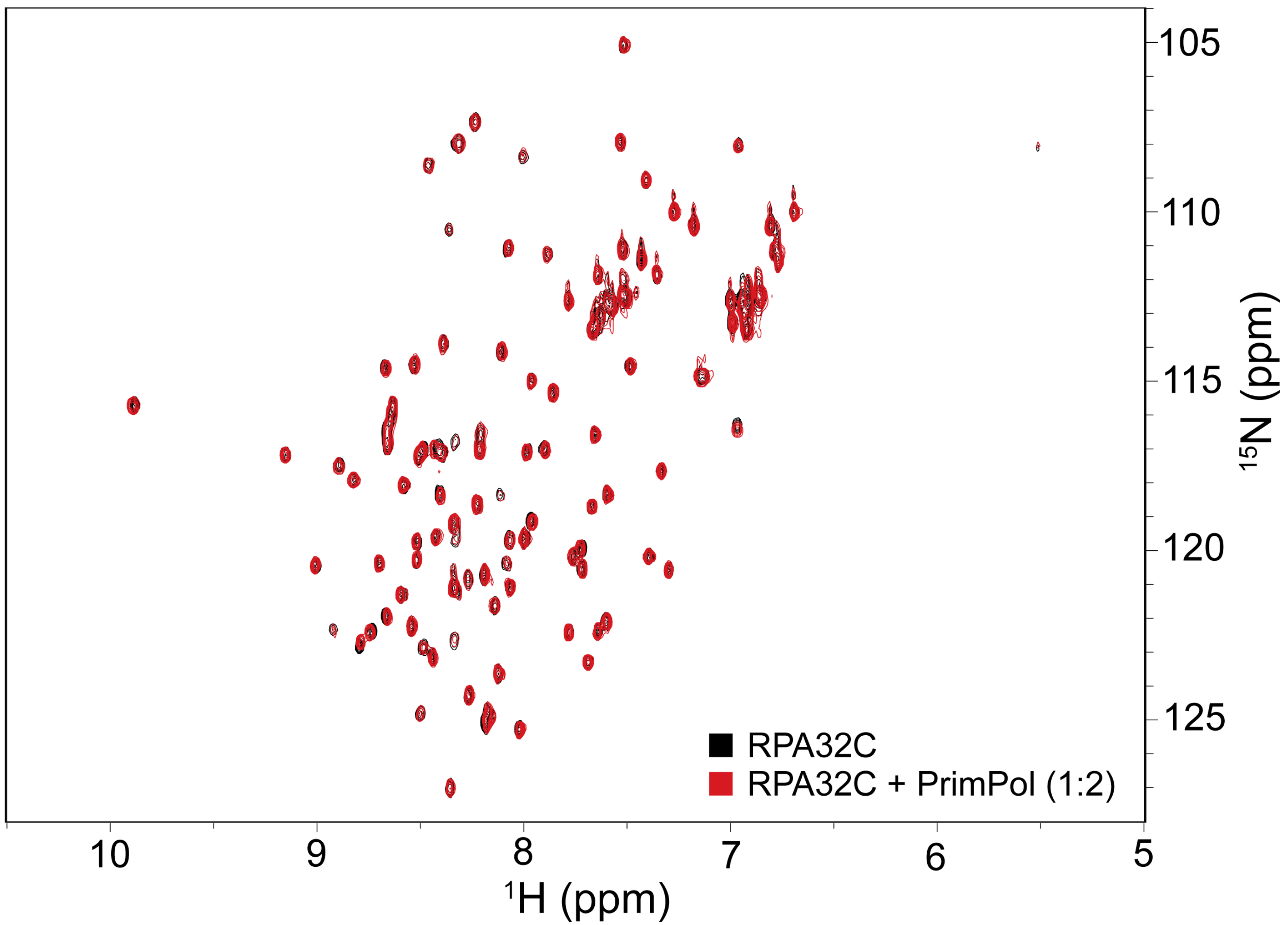

Fig. S2. NMR titration of RPA32C with PrimPol

^{15}N-^{1}H HSQC overlay of ^{15}N-enriched RPA32C alone (black) or in the presence of two-fold molar excess of PrimPol (red).

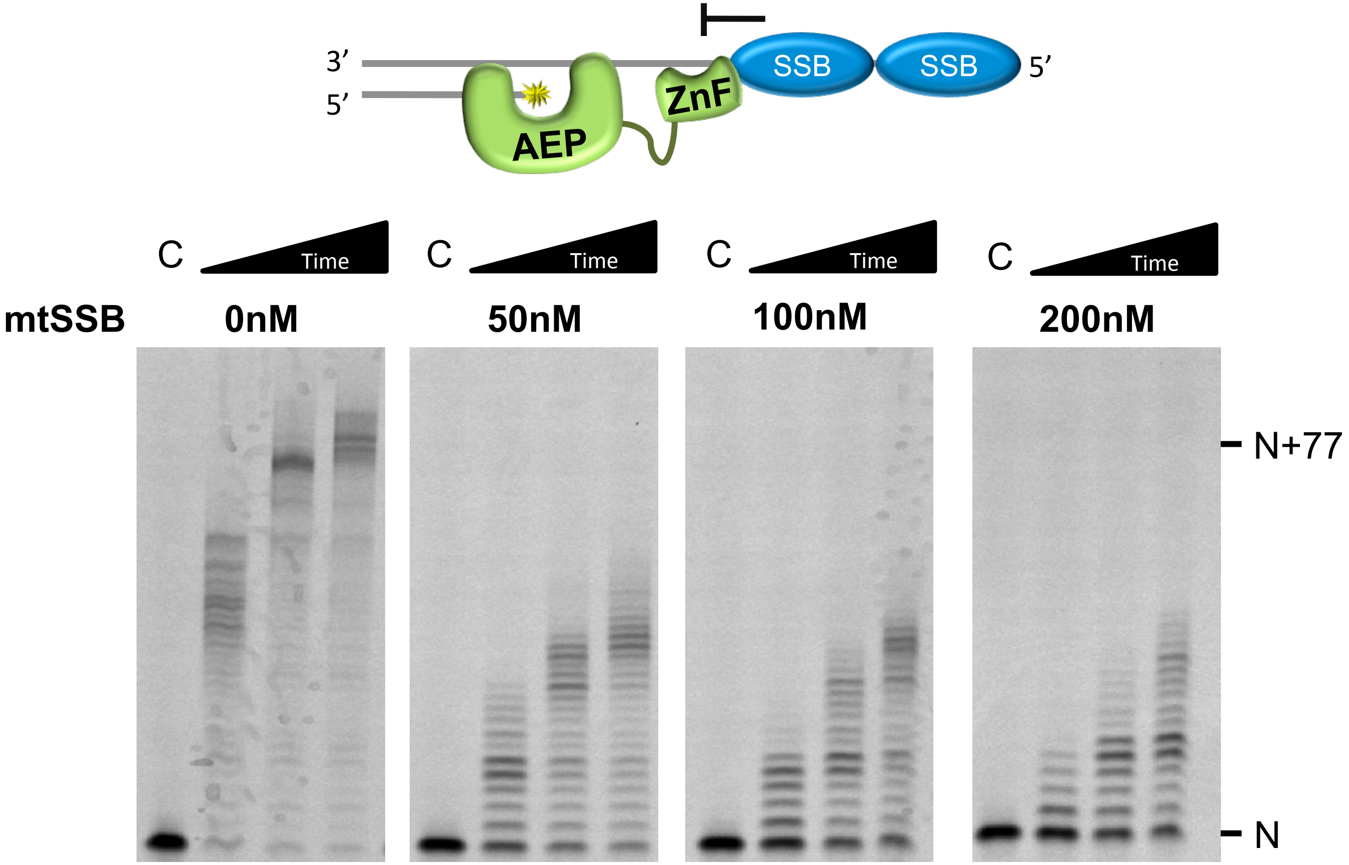

Fig. S3. mtSSB inhibits PrimPol over a large range of protein concentrations
The concentration of mtSSB in each assay is indicated above each gel. The time-points used are 1min, 5min, and 10min.

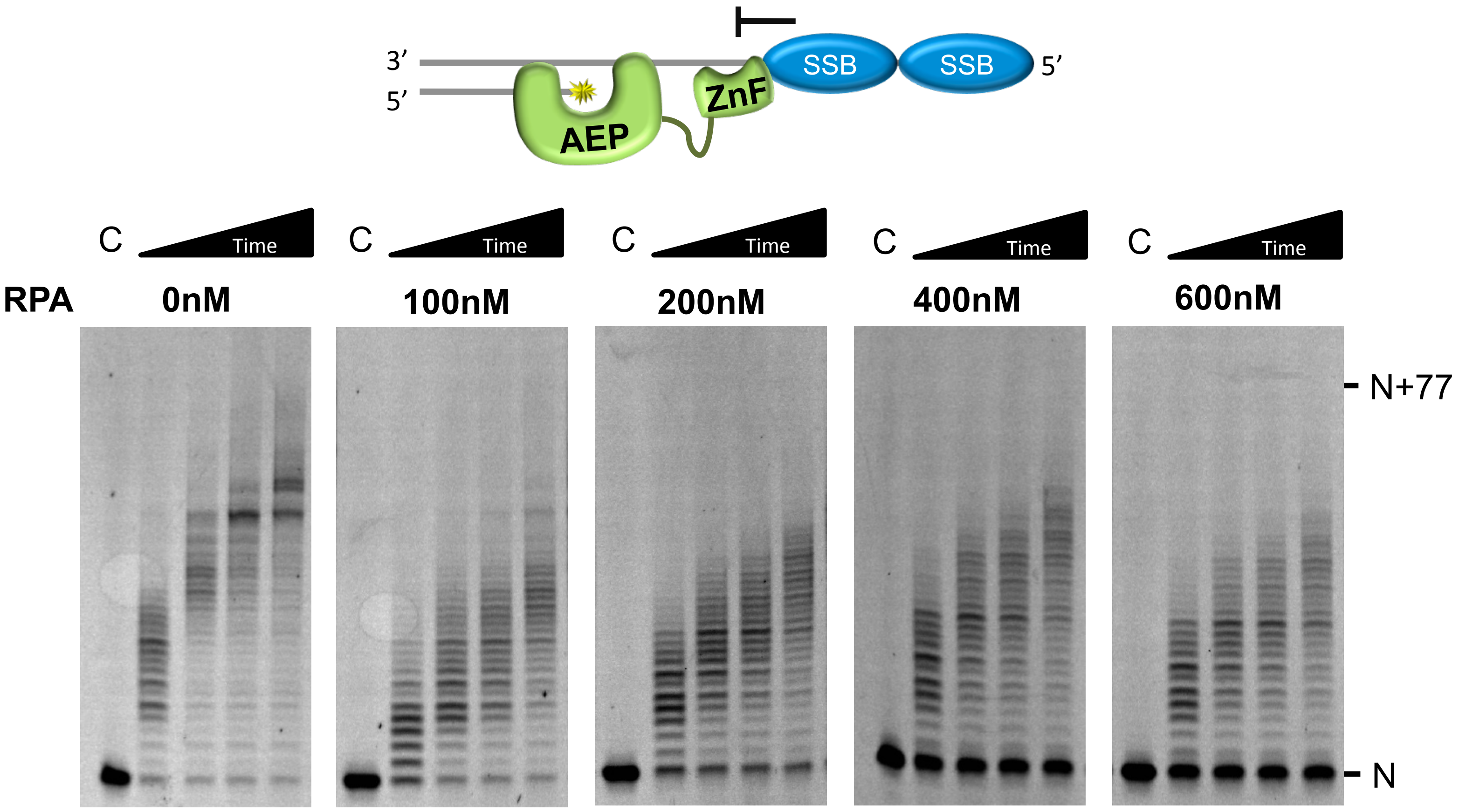

Fig. S4. RPA inhibits PrimPol over a large range of protein concentrations
The concentration of RPA in each assay is indicated above each gel. 'C' indicates the no enzyme control.

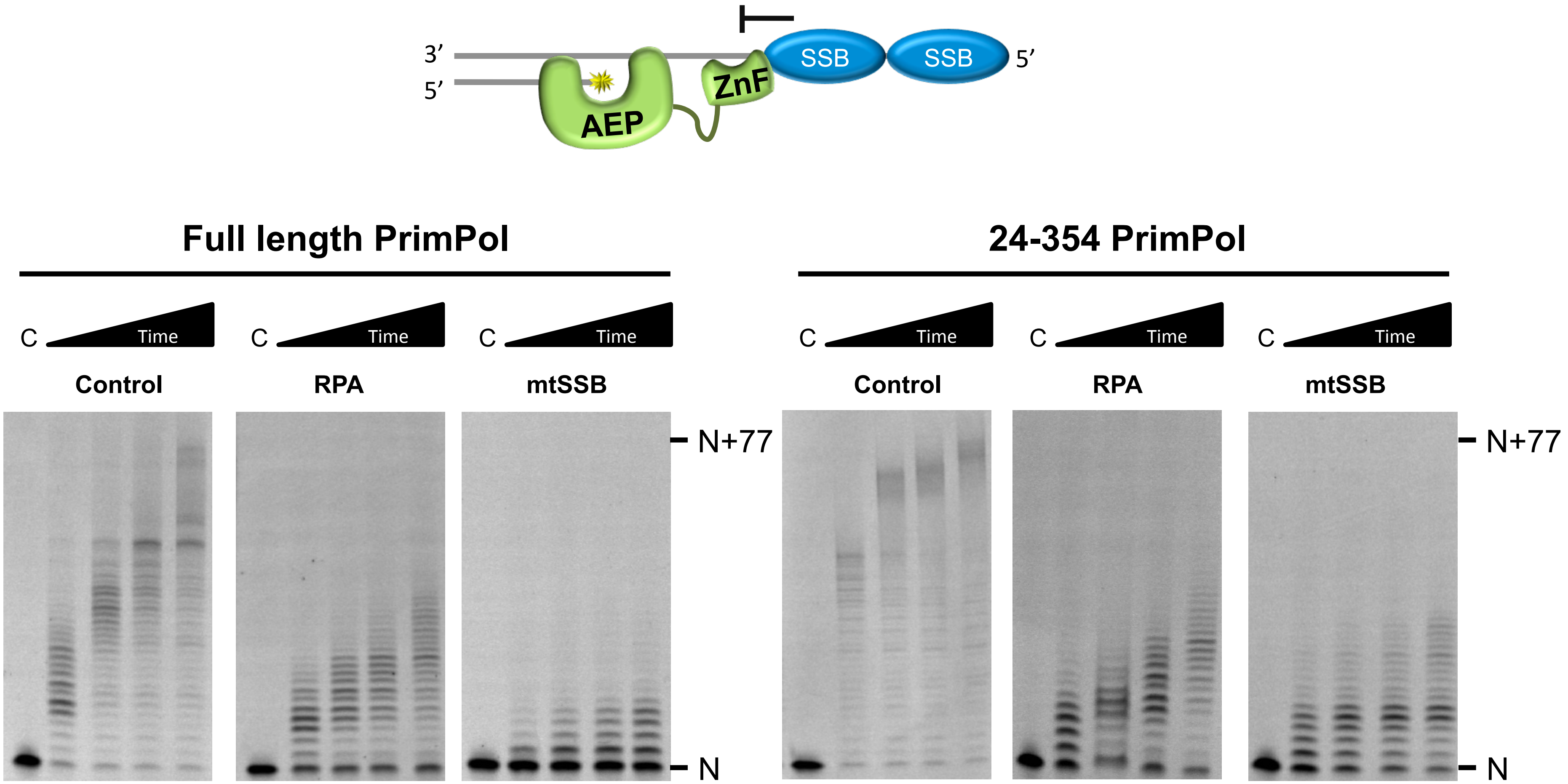

Fig. S5. SSBs inhibit PrimPol even when the enzyme is pre-incubated with DNA

Full-length and 24-354 PrimPol were pre-incubated with the DNA template to allow binding before the addition of dNTPs and either RPA or mtSSB. In each case inhibition of primer extension was observed. ‘C’ indicates the no enzyme control.

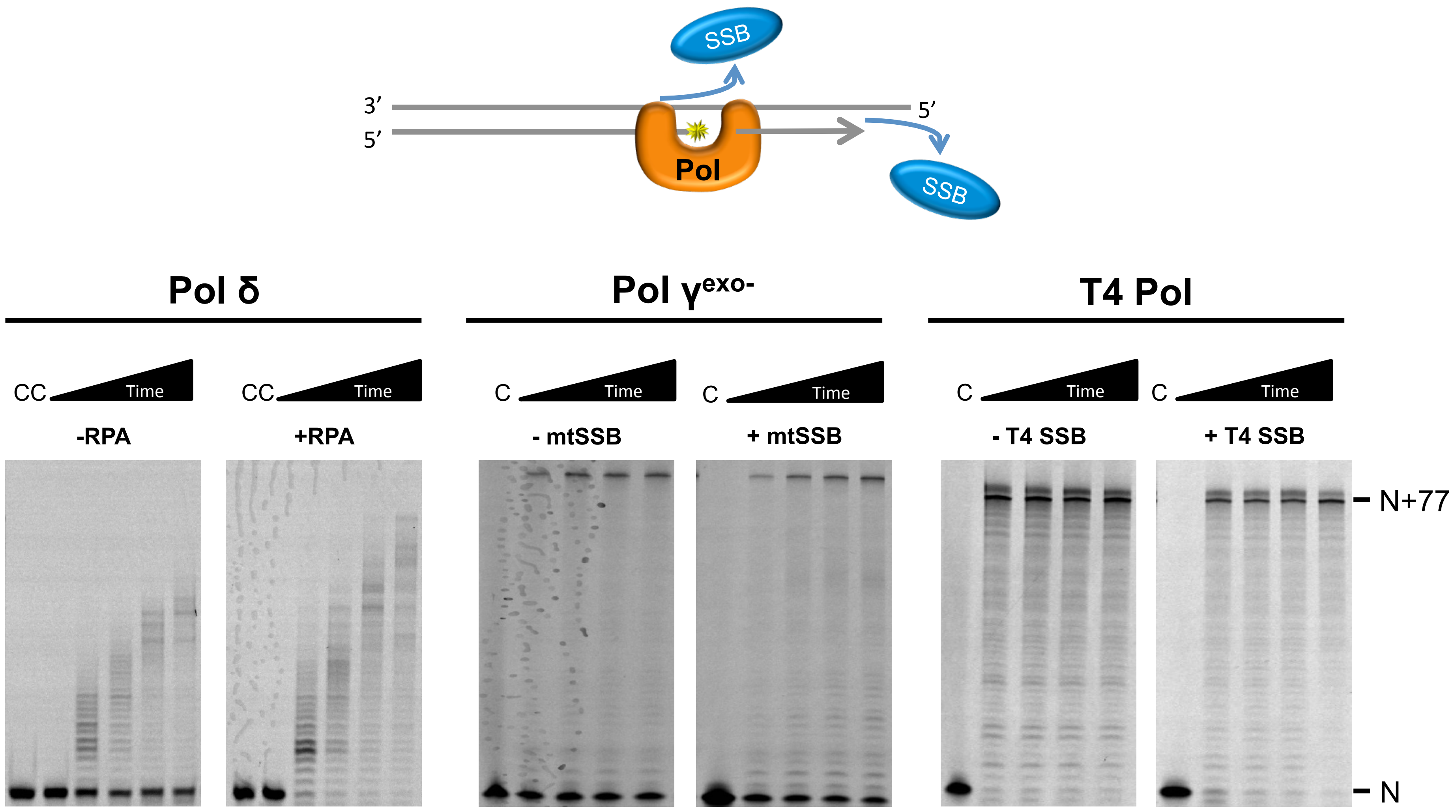

Fig. S6. Replicative polymerases are able to displace SSBs. Polδ with PCNA, Polγ^{exo-}, and T4 Pol, are able to displace RPA, mtSSB, and T4 SSB, from DNA, respectively. 'C' indicates the no enzyme control.

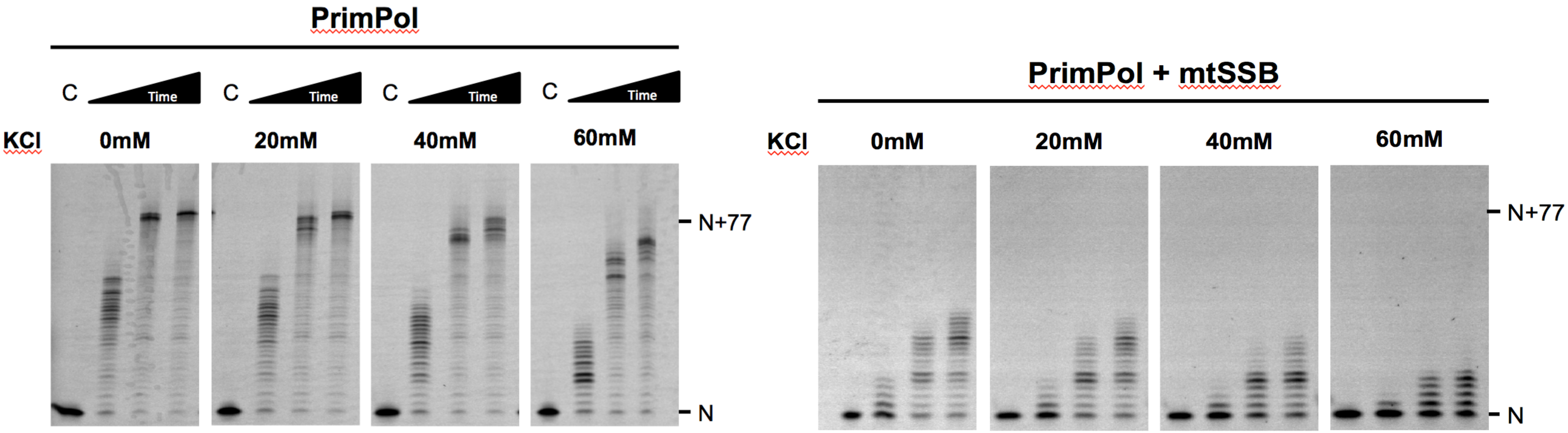

Fig. S7. PrimPol is inhibited by mtSSB over a large range of salt concentrations
The concentration of KCl in the buffer for each assay is indicated above each gel. The time-points used are 1min, 5min, and 10min. 'C' indicates the no enzyme control.

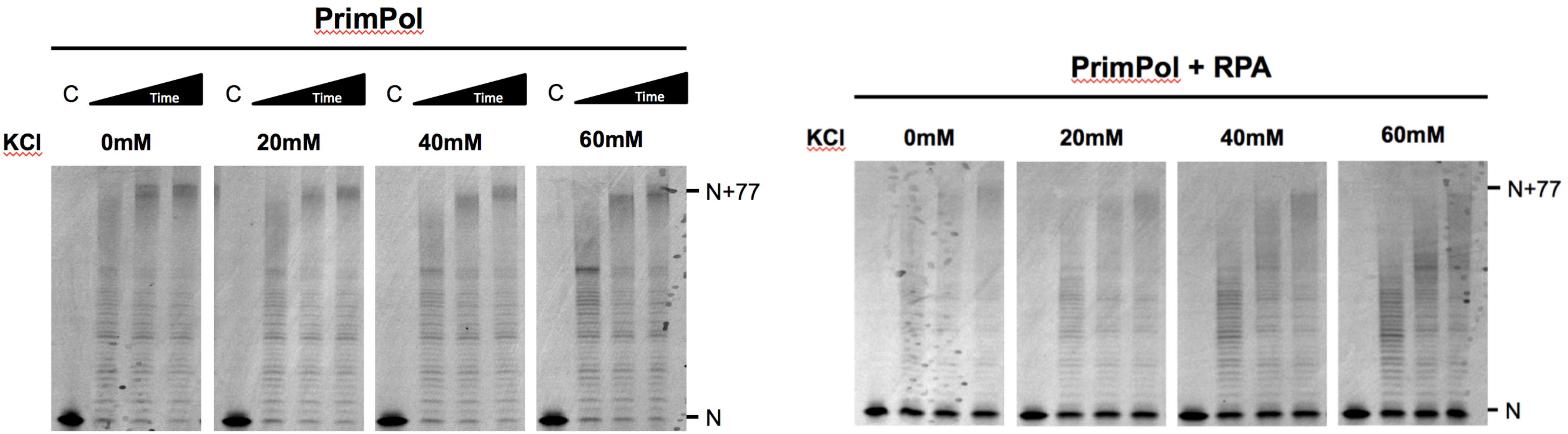

Fig. S8. PrimPol is inhibited by RPA over a large range of salt concentrations.
The concentration of KCl in the buffer for each assay is indicated above each gel.
The time-points used are 1min, 5min, and 10min. 'C' indicates the no enzyme control.

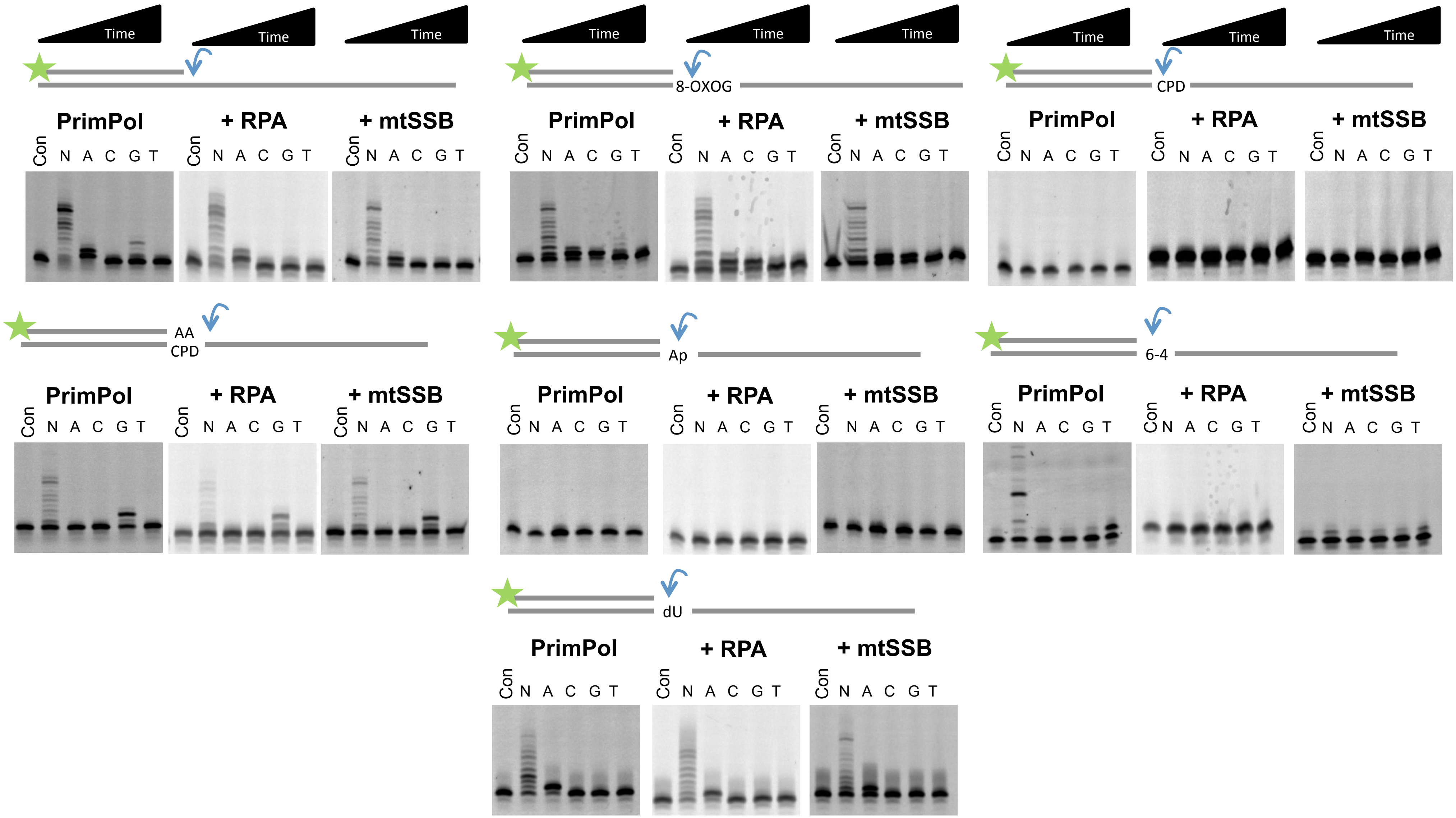

Fig. S9. PrimPol HSV-tk coding region error spectrum. Base substitutions are shown above the template sequence, highlighted green for detectable events or yellow for non-detectable. Single deletion and insertion events are shown below the template with open and closed triangles respectively, while diamonds indicate a tandem deletion. Superscripts mark the errors found within an individual complex event. Long lines under the template sequence note >2nt deletion events.

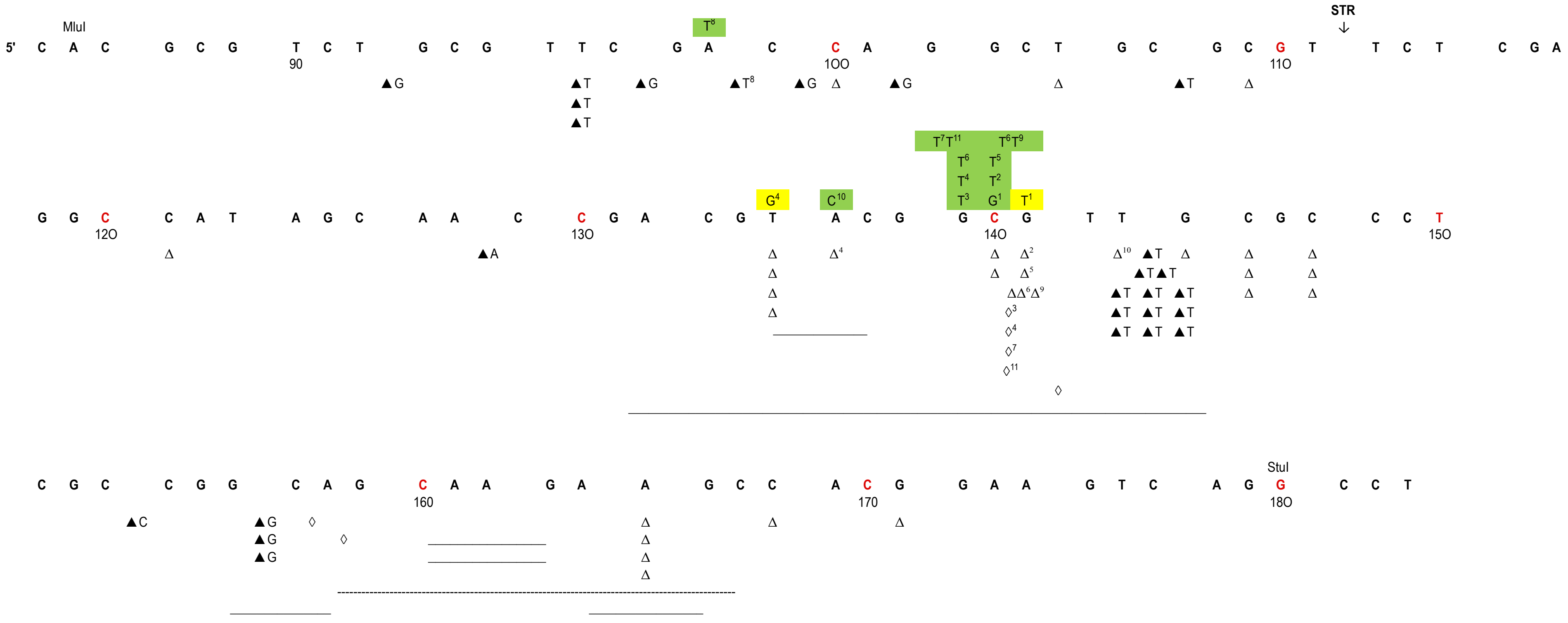

Fig. S10. PrimPol HSV-tk coding region error spectrum

Base substitutions are shown above the template sequence, highlighted green for detectable events or yellow for non-detectable. Single deletion and insertion events are shown below the template with open and closed triangles respectively, while diamonds indicate a tandem deletion. Superscripts mark the errors found within an individual complex event. Long lines under the template sequence note >2nt deletion events.

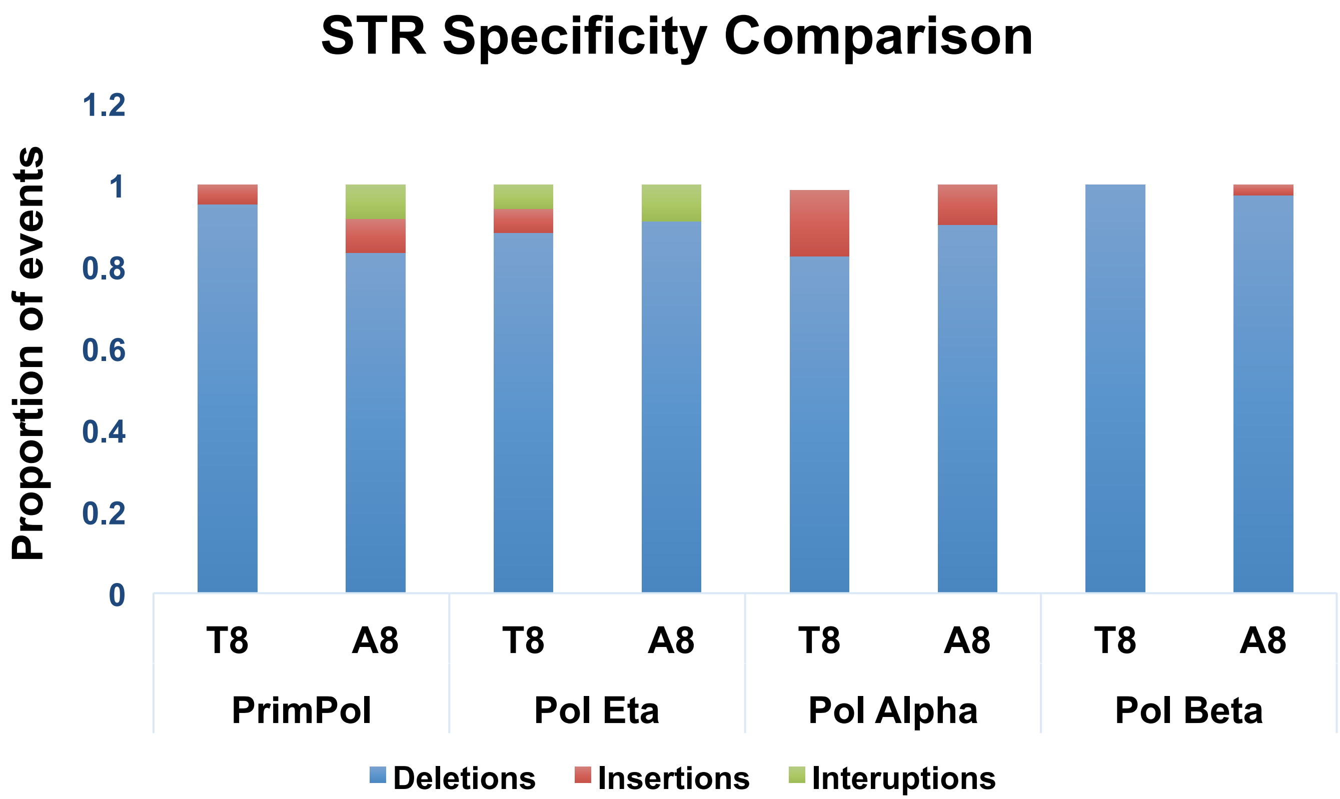

Fig. S11. The STR is not driving PrimPol's elevated mutation frequency
Error specificity at the STR is shown for PrimPol in comparison to the other polymerases we have examined on both templates.
